# The NLRP3 Inflammasome: Mechanisms of Activation, Regulation, and Therapeutic Opportunities

**DOI:** 10.1002/mco2.70660

**Published:** 2026-03-05

**Authors:** Chan Zou, Shilong Jiang, Hui Li, Kai Zhao, Dongshen Cao, Guoping Yang

**Affiliations:** ^1^ Center For Clinical Pharmacology Third Xiangya Hospital Central South University Changsha China; ^2^ Department of Pharmacy Xiangya Hospital Central South University Changsha China; ^3^ The Hunan Institute of Pharmacy Practice and Clinical Research Changsha China; ^4^ National Clinical Research Center for Geriatric Disease Xiangya Hospital Central South University Changsha China; ^5^ Xiangya School of Pharmaceutical Science Central South University Changsha China; ^6^ Department of Hematology and Critical Care Medicine the Third Xiangya Hospital Central South University Changsha China

**Keywords:** activation mechanisms, inflammatory diseases, innate immunity, NLRP3 inflammasome, therapeutic inhibitors

## Abstract

The NLRP3 inflammasome is a pivotal signaling platform of the innate immune system that senses a broad spectrum of microbial, metabolic, and environmental danger signals. Its activation leads to the recruitment of ASC and caspase‐1, driving the maturation of pro‐inflammatory cytokines interleukin (IL)‐1β and IL‐18 as well as the execution of pyroptosis. Aberrant or persistent activation of NLRP3 has been implicated in the pathogenesis of numerous disorders, including autoinflammatory syndromes, metabolic and cardiovascular diseases, neurodegenerative conditions, and cancers. In this review, we provide an updated overview of the molecular mechanisms governing NLRP3 activation and regulation, with particular focus on ion flux, mitochondrial damage, lysosomal rupture, reactive oxygen species, and post‐translational modifications. We further discuss negative regulatory pathways that maintain inflammasome homeostasis and prevent excessive inflammation. Finally, we summarize recent advances in therapeutic strategies targeting the NLRP3 inflammasome, ranging from direct inhibitors and allosteric modulators to biologics and repurposed drugs, and highlight their translational potential. Understanding the fine balance between NLRP3 activation and inhibition offers new opportunities for therapeutic intervention in a wide array of inflammatory and immune‐related diseases.

## Graphical abstract

Diverse diseases converge on NLRP3. We depict a discovery‐to‐clinic track: high‐throughput/phenotypic screens, structure‐guided design, and modality innovation (allosteric inhibitors, interface blockers, degraders) deliver third‐generation, disease‐tailored NLRP3 control. Mechanistic insights into activation and regulation map druggable nodes to indications, enabling precision matching of compounds to patient contexts.

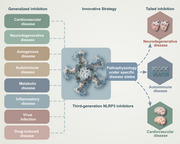

## 1 Introduction

The innate immune system is the body's first line of defense against infection and injury. It relies on pattern recognition receptors (PRRs) to detect pathogen‐associated molecular patterns (PAMPs) and danger‐associated molecular patterns (DAMPs) [1]. Among the key PRRs are Toll‐like receptors (TLRs), retinoic acid‐inducible gene‐I‐like receptors (RLRs), and nucleotide‐binding oligomerization domain (NOD)‐like receptors (NLRs) [2]. NLRs are highly conserved and comprise at least 23 members in humans, divided into five subfamilies based on their N‐terminal domains: NLRA, NLRB, NLRC, NLRP, and NLRX [3]. The identification of NLRP3 as the causal gene for cryopyrin‐associated periodic syndromes (CAPS) in 2001 marked a major milestone, linking NLRP3 mutations to excessive interleukin (IL)‐1β production and systemic inflammation [4].

The NLRP3 inflammasome functions as a cytosolic sensor that integrates a wide range of microbial and sterile danger signals. Its activation generally follows a two‐step model, comprising a priming phase that upregulates NLRP3 and pro‐inflammatory cytokines, and an activation phase in which diverse perturbations initiate inflammasome assembly and downstream signaling [5]. Beyond this canonical pathway, non‐canonical and alternative routes further expand the contexts in which NLRP3 can be engaged [6, 7, 8, 9]. Structural and biochemical advances have revealed that NLRP3 exists in an autoinhibited cage‐like conformation, requiring NEK7‐mediated licensing and ATP‐dependent oligomerization to form active inflammasomes. This structural flexibility underscores NLRP3's dual nature—highly responsive yet stringently controlled—to balance defense and immune tolerance.

**FIGURE 1:**
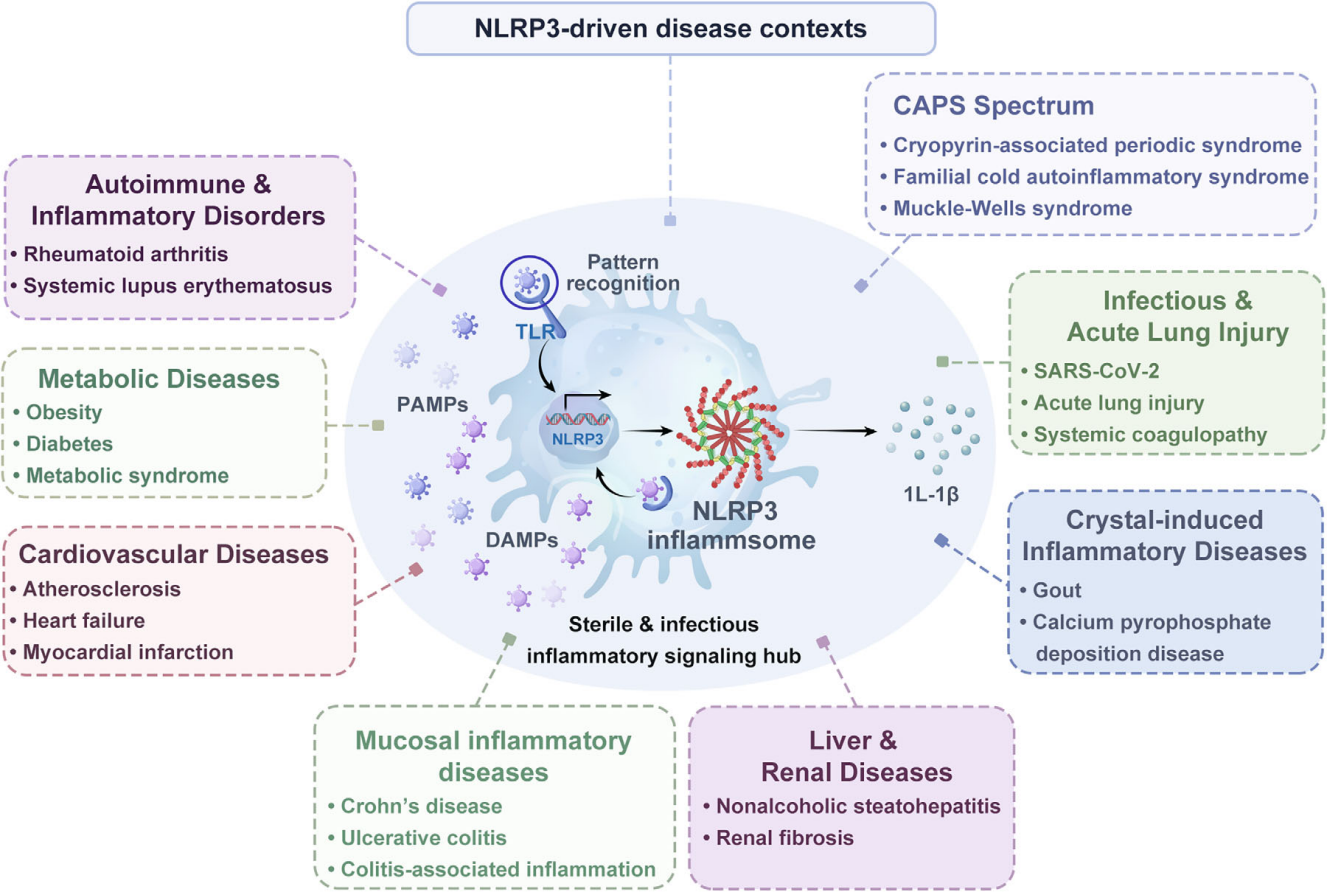
NLRP3‐driven disease contexts.Schematic overview showing NLRP3 inflammasome as a sterile‐ and infection‐associated inflammatory signaling hub activated by pattern recognition and danger cues, contributing to diverse disease settings, including autoinflammatory disorders (CAPS spectrum), autoimmune/inflammatory disorders, metabolic diseases, cardiovascular diseases, mucosal inflammatory diseases, liver/renal diseases, infectious diseases, and acute lung injury, and crystal‐induced inflammatory diseases. CAPS, cryopyrin‐associated periodic syndromes; DAMPs, damage‐associated molecular patterns; IL‐1β, interleukin‐1 beta; NLRP3, NOD‐like receptor family pyrin domain containing 3; PAMPs, pathogen‐associated molecular patterns; SARS‐CoV‐2, severe acute respiratory syndrome coronavirus 2; TLR, Toll‐like receptor.

Aberrant or sustained activation of the NLRP3 inflammasome has been implicated in a wide spectrum of diseases, including autoimmune and metabolic disorders, cardiovascular pathologies, and neurodegenerative conditions (Figure 1). Its central role as a convergence point for sterile inflammation has rendered NLRP3 a compelling therapeutic target. Over the past decade, intensive efforts have been devoted to the discovery of small‐molecule inhibitors, leading from early probes such as MCC950 to multiple clinical candidates currently in phase I–III trials. Parallel breakthroughs in structural biology, immunometabolism, and chemical biology have deepened understanding of NLRP3's activation logic and provided diverse pharmacological entry points.

This review aims to provide a comprehensive and integrative understanding of the NLRP3 inflammasome, emphasizing how its molecular activation mechanisms, multilayered regulatory circuits, and therapeutic interventions form an interconnected framework. We first discuss the molecular logic that governs NLRP3 activation, including structural licensing, organellar communication, and metabolic integration. We then examine the regulatory mechanisms—ranging from transcriptional and post‐translational control to proteostatic and metabolic modulation—that maintain inflammasome responsiveness and prevent excessive activation. Finally, we summarize current progress in the discovery and optimization of NLRP3 inhibitors, outlining representative chemical scaffolds, discovery paradigms, and translational developments across various inflammatory diseases. Together, these discussions provide an updated conceptual synthesis linking the mechanistic foundations of NLRP3 biology to emerging therapeutic strategies.

## 2 Mechanisms of NLRP3 Inflammasome Activation

**FIGURE 2:**
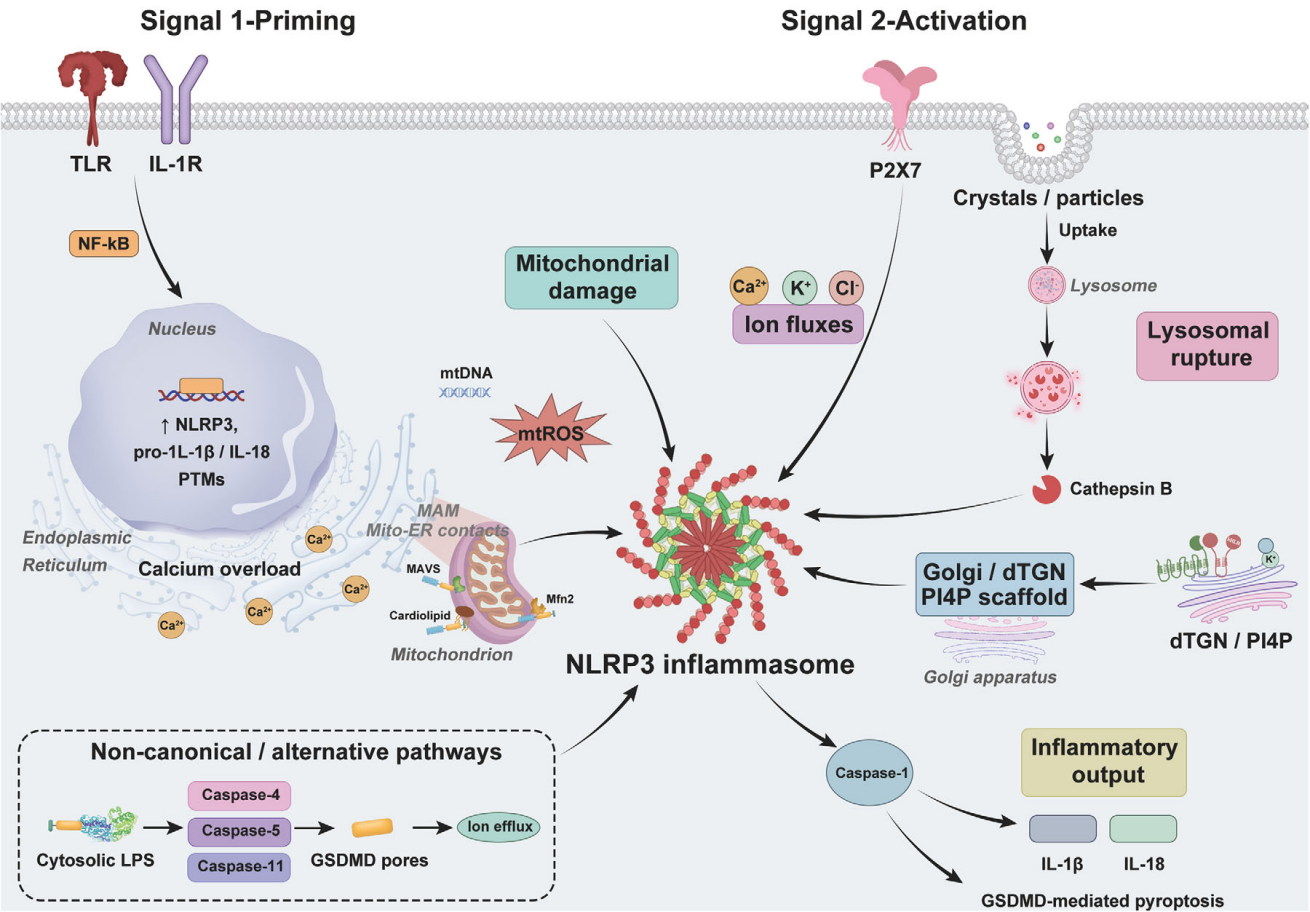
Two‐signal model of NLRP3 inflammasome activation and alternative pathways. Signal 1 (priming) initiated by receptors (e.g., TLR/IL‐1R) activates NF‐κB to upregulate NLRP3 and pro‐IL‐1β/pro‐IL‐18 and to shape permissive PTMs. Signal 2 (activation) integrates diverse cellular stress events—ion fluxes, mitochondrial damage (mtROS/mtDNA), lysosomal rupture with cathepsin release, and Golgi/dTGN PI4P scaffolding—to promote NLRP3 inflammasome assembly, caspase‐1 activation, cytokine maturation, and GSDMD‐mediated pyroptosis. Non‐canonical inflammasome activation via cytosolic LPS activates caspase‐4/5 (human) or caspase‐11 (mouse), inducing GSDMD pore formation and secondary NLRP3 activation. dTGN, dispersed trans‐Golgi network; GSDMD, gasdermin D; IL‐1β, interleukin‐1 beta; IL‐18, interleukin‐18; IL‐1R, interleukin‐1 receptor; LPS, lipopolysaccharide; MAM, mitochondria‐associated membranes; MAVS, mitochondrial antiviral‐signaling protein; Mfn2, mitofusin 2; mtDNA, mitochondrial DNA; mtROS, mitochondrial reactive oxygen species; NF‐κB, nuclear factor kappa B; NLRP3, NOD‐like receptor family pyrin domain containing 3; P2×7, purinergic receptor P2×7; PI4P, phosphatidylinositol 4‐phosphate; PTMs, post‐translational modifications; TLR, Toll‐like receptor.

NLRP3 inflammasome activation is a multi‐layered process that integrates diverse extracellular and intracellular signals into a unified inflammatory response. This section summarizes the stepwise mechanisms underlying its activation — from canonical ionic and particulate triggers to mitochondrial and metabolic integration, structural licensing, and non‐canonical pathways. Together, these interconnected mechanisms explain how distinct stimuli converge on a shared molecular architecture to initiate inflammasome assembly and cytokine maturation (Figure 2).

### 2.1 Canonical Activation (Proximal Triggers)

The canonical NLRP3 inflammasome is activated through a well‐established two‐signal model. The “priming” signal is mediated by Toll‐like receptors (TLRs), cytokine receptors, or other nuclear factor kappa B (NF‐κB)–activating pathways, which induce transcriptional upregulation of NLRP3, pro‐IL‐1β, and pro‐IL‐18, and introduce post‐translational modifications (PTMs) that set the activation threshold [10, 11]. After priming, a second activation signal arises from proximal cellular perturbations that license assembly.

A fall in cytosolic K^+^ is a unifying cue for NLRP3 activation, typically driven by P2×7 engagement and, in macrophages, by the K2P channel TWIK2, which couples extracellular ATP to K^+^ efflux and inflammasome activation [12, 13, 14, 15]; In parallel, Ca^2^^+^ mobilization from ER stores and extracellular sources promotes mitochondrial dysfunction and ROS production, thereby facilitating assembly [16, 17]; Cl^−^ efflux via CLIC1/CLIC4, which operates upstream to foster the NEK7–NLRP3 interaction and lower the activation threshold, integrating with K^+^/Ca^2^^+^ signals [18, 19]. Together, these ionic disturbances provide a rapid, conserved licensing layer for inflammasome assembly.

Beyond ionic fluxes, lysosomal destabilization represents a particulate/crystal‐triggered proximal event. Crystalline or aggregated materials (e.g., silica, alum, and cholesterol crystals) are internalized and damage endo‐lysosomes; ensuing lysosomal membrane permeabilization/rupture releases cathepsins (notably cathepsin B), which promote NLRP3 assembly and IL‐1β maturation in multiple settings [20, 21, 22]. This pathway explains how sterile particles provide a cytosolic danger context converging on the inflammasome.

Certain agonists also induce rapid membrane‐platform remodeling that licenses assembly in a parallel, proximal manner. Specifically, trans‐Golgi network (TGN) disassembly generates PI4P‐rich dispersed TGN (dTGN) membranes that recruit and cluster NLRP3 through a polybasic region–PI4P interaction; this routing is functionally coupled to PKD‐dependent phosphorylation at the Golgi and can operate even when mitochondrial cues are limited, thereby acting as an early, common scaffold upstream of full inflammasome assembly [23, 24, 25].

In context‐specific scenarios (e.g., hypotonic stress), LRRC8A‐containing VRAC channels provide an anion/volume‐regulatory route that licenses NLRP3 activation; importantly, this mechanism appears selective for hypotonicity and is dispensable for many DAMP‐driven stimuli, so it is considered an optional proximal input rather than a universal requirement [26]. These proximal signals converge on common events that destabilize ionic gradients and specific membrane platforms, ultimately licensing NLRP3 oligomerization, ASC filament formation, caspase‐1 activation, and the maturation of IL‐1β/IL‐18 [5, 27, 28, 29]. (Mitochondrial danger signals and metabolic/organellar integration are detailed in 2.2).

### 2.2 Emerging Modulators: Mitochondrial, Metabolic, and Organellar Regulation

Beyond the immediate licensing events, mitochondria and cellular metabolism act as integrators/amplifiers that shape the magnitude and persistence of activation. Mitochondrial dysfunction generates signals—mtROS via reverse electron transport (RET) at complex I, oxidized mtDNA release through permeability transition, and cardiolipin externalization—each capable of engaging NLRP3 directly or indirectly [30, 31]. RET‐derived mtROS act as selective signals for IL‐1β release in inflammatory macrophages, coupling bioenergetics with cytokine output [30]. Defective mitophagy, particularly parkin/PINK1‐dependent clearance, leads to accumulation of these signals and sustained inflammasome activity [32].

Metabolic reprogramming further fine‐tunes NLRP3 activation. Enhanced glycolysis, mediated by PKM2 and the HK1–mTORC1 axis, promotes lactate‐EIF2AK2 signaling and transcriptional induction of glycolytic genes [33, 34], whereas high glucose conditions amplify inflammasome activation through MARK4‐dependent microtubule dynamics and increased mtROS [35, 36]. Within the tricarboxylic acid cycle, succinate accumulation under hypoxic stress stabilizes HIF‐1α to promote NLRP3 activation [37, 38], while itaconate, produced by IRG1, acts as an endogenous inhibitor by covalently modifying NLRP3 and disrupting NEK7 binding [39, 40]. Lipid metabolism exerts bidirectional control: saturated fatty acids such as palmitate induce K^+^ efflux and mtROS generation [41], whereas ketone bodies like β‐hydroxybutyrate and microbial short‐chain fatty acids suppress inflammasome signaling, linking host diet and gut microbiota to immune regulation [42, 43]. Notably, recent work identifies lactylation as a glycolysis‐driven PTM that bridges metabolism and NLRP3 control—either by directly modifying inflammasome components or via histone lactylation/ALKBH5‐m6A axes in silica injury and AKI‐to‐CKD transition models [44, 45].

Recent studies highlight a tight coupling between carbon metabolism and canonical NLRP3 activation. When glycolysis/energy metabolism is blocked (a “carbon starvation” state), canonical caspase‐1 activation and IL‐1β/IL‐18 maturation are suppressed; however, this metabolic constraint can instead precipitate a distinct, mitochondria‐dependent and ROS‐amplified non‐pyroptotic lytic death termed mitoxyperiosis, which may reshape innate–adaptive immune outcomes during infection [46, 47].

Beyond mitochondria and metabolism, organelle‐specific mechanisms contribute additional layers: ER stress sensors (IRE1, PERK, and ATF6) can couple proteostasis stress to inflammasome readiness, while Golgi–endosome trafficking and spatiotemporal relocalization (e.g., dTGN scaffolds referenced in 2.1) modulate the efficiency and locality of assembly [48]. How these organelles and membranes *position* NLRP3 to licensing sites—and how trafficking programs interface with assembly competence—is discussed in Section 3.3 (spatial & proteostatic control). Moreover, Apolipoprotein C3 (ApoC3) can potentiate sterile inflammasome signaling through TLR2/4–SCIMP–SYK‐linked calcium influx and ROS production, reinforcing alternative/monocyte‐biased routes [49].

### 2.3 Structural Licensing and Inflammasome Assembly

**FIGURE 3:**
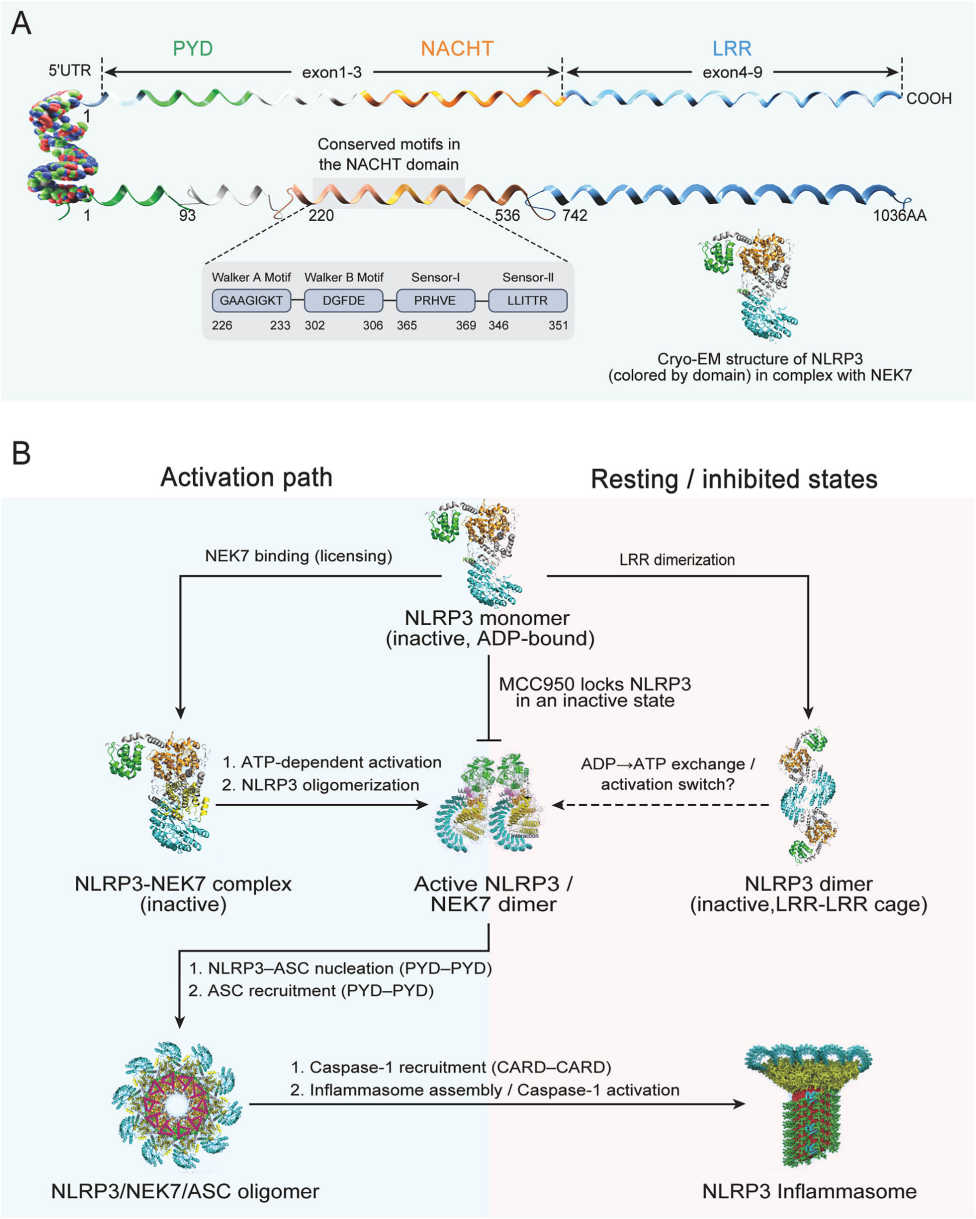
Structural basis and conformational transitions of NLRP3 activation and inhibition. (A) Domain architecture of NLRP3, highlighting PYD–NACHT–LRR organization and conserved NACHT motifs, together with a representative cryo‐EM structure of NLRP3 in complex with NEK7. (B) Proposed conformational landscape from inactive NLRP3 monomer to NEK7‐licensed states, ATP‐dependent activation/oligomerization, ASC nucleation and recruitment, caspase‐1 engagement, and mature inflammasome assembly; resting/inhibited states and MCC950‐mediated stabilization of an inactive conformation are indicated. ADP, adenosine diphosphate; ASC, apoptosis‐associated speck‐like protein containing a CARD; ATP, adenosine triphosphate; CARD, caspase activation and recruitment domain; Cryo‐EM, cryo‐electron microscopy; LRR, leucine‐rich repeats; NACHT, NAIP/C2TA/HET‐E/TP1 domain; NEK7, NIMA‐related kinase 7; NLRP3, NOD‐like receptor family pyrin domain containing 3; PYD, pyrin domain; UTR, untranslated region.

NLRP3 is a tripartite protein composed of an N‐terminal pyrin domain (PYD), a central nucleotide‐binding and oligomerization domain (NACHT), and a C‐terminal leucine‐rich repeat (LRR) domain [5, 50] (Figure 3). The PYD mediates homotypic interactions with the adaptor protein ASC, which nucleates filamentous assemblies essential for inflammasome formation [5]. The NACHT domain contains multiple subdomains—including the nucleotide‐binding domain (NBD), helical domains (HD1 and HD2), the winged‐helix domain (WHD), and the FISNA domain—which coordinate ATP‐dependent conformational changes and oligomerization [51, 52]. The LRR domain contributes to autoinhibition, ligand recognition, and recruitment of the regulatory kinase NEK7, a critical licensing factor for NLRP3 activation [53]. Notably, the FISNA subdomain is absent in most other NLR proteins, highlighting evolutionary specialization of NLRP3 for precise structural regulation [54].

In resting cells, cryo‐EM studies show NLRP3 adopts a closed, cage‐like oligomer stabilized by LRR‐LRR interactions, serving as a metastable autoinhibited reservoir [55, 56]. Licensing by NEK7—structurally captured in the NEK7–NLRP3 complex—relieves this autoinhibition by binding the LRR and weakening LRR‐NACHT constraints, thereby permitting NACHT‐mediated oligomerization [53].

Upon licensing, ATP/ADP exchange at NACHT and a characteristic hinge rotation shift NLRP3 into an active conformation that assembles into planar, wheel‐like “disc” oligomers; the active disc architecture has been resolved at near‐atomic resolution and provides a structural basis for ASC recruitment [51]. ASC is then recruited via PYD–PYD interactions to nucleate helical filaments, which in turn engage pro‐caspase‐1 through CARD–CARD contacts, enabling caspase‐1 dimerization, autocatalysis, and downstream processing of pro‐IL‐1β, pro‐IL‐18, and GSDMD. Gain‐of‐function mutations (e.g., CAPS) destabilize the autoinhibited state and lower the activation threshold, driving constitutive activity [57, 58, 59]. These structural insights have informed therapeutic strategies; for instance, MCC950 stabilizes the inactive NACHT conformation and prevents oligomerization [60].

### 2.4 Non‐Canonical and Alternative Pathways

Distinct non‐canonical and alternative pathways expand the repertoire of NLRP3 activation. The non‐canonical pathway is initiated when cytosolic lipopolysaccharide (LPS) is sensed directly by caspase‐4 and caspase‐5 in humans (or caspase‐11 in mice), independent of TLR4 [61, 62]. Upon LPS binding, these caspases cleave gasdermin D (GSDMD), forming membrane pores that drive ionic efflux and pyroptosis [63]. These cellular disturbances in turn activate NLRP3, highlighting its role as a secondary amplifier of non‐canonical inflammasome signaling [64]. The alternative pathway, first described in human monocytes, is distinct from both canonical and non‐canonical activation. Here, prolonged LPS stimulation engages the TLR4–TRIF–RIPK1–FADD–caspase‐8 axis, leading to NLRP3 activation and IL‐1β release in a manner independent of potassium efflux or ASC speck formation [65]. Recent evidence also indicates that apolipoprotein C3 (ApoC3) can potentiate this pathway by interacting with TLR2/4, promoting calcium influx and ROS production to reinforce inflammasome signaling [66, 67]. These findings underscore NLRP3's role as a versatile integrator beyond classical microbial or crystalline stimuli.

## 3 Regulatory Mechanisms of the NLRP3 Inflammasome

**FIGURE 4:**
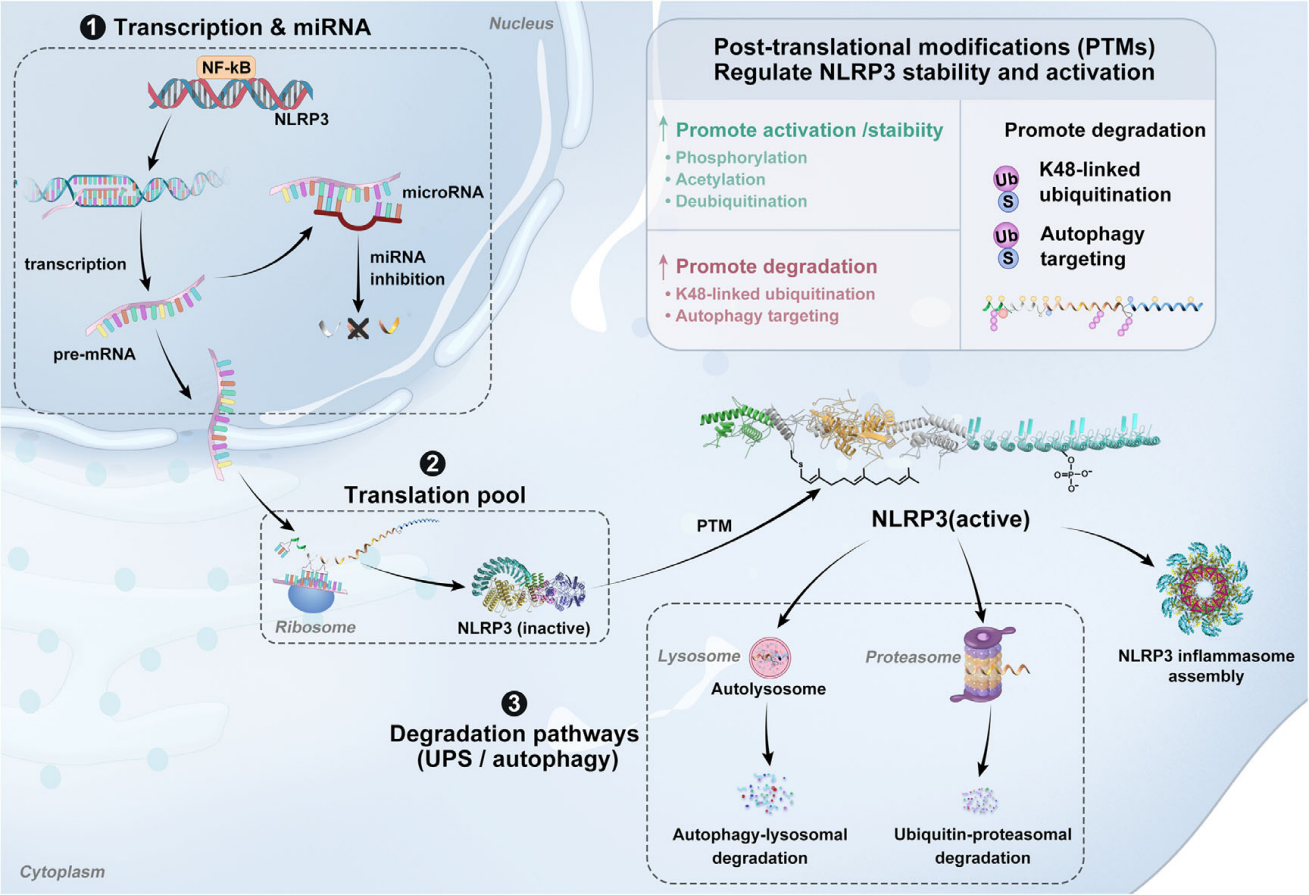
Multilayer regulation of NLRP3 abundance, activation, and turnover. NLRP3 activity is regulated at multiple levels: (1) transcriptional control (e.g., NF‐κB–dependent induction) and post‐transcriptional modulation by microRNAs; (2) protein translation and formation of the cytosolic NLRP3 pool; and (3) post‐translational modifications that tune NLRP3 stability/activation versus degradation, with clearance mediated by ubiquitin–proteasome and autophagy–lysosome pathways. K48, lysine‐48–linked ubiquitination; miRNA, microRNA; NF‐κB, nuclear factor kappa B; NLRP3, NOD‐like receptor family pyrin domain containing 3; PTMs, post‐translational modifications; Ub, ubiquitin; UPS, ubiquitin–proteasome system.

The activity of the NLRP3 inflammasome is tightly controlled through multiple, interdependent regulatory mechanisms that operate at the transcriptional, post‐transcriptional, and metabolic levels, ensuring an appropriate immune response while preventing excessive inflammation [68]. These regulatory mechanisms collectively determine the threshold, magnitude, and duration of inflammasome activation, providing multiple therapeutic targets for intervention in inflammatory diseases (Figure 4).

### 3.1 Transcriptional and Epigenetic Priming

Transcriptional priming sets the size and readiness of the NLRP3/pro‐IL‐1β/pro‐IL‐18 pool [69]. The canonical NF‐κB pathway is the primary driver of transcriptional priming, with RelA/p65 binding κB sites within the NLRP3 promoter [10]. Metabolic and environmental cues further influence transcription: hypoxia stabilizes HIF‐1α to enhance NLRP3 expression [37, 38], whereas Nrf2 exerts dual, context‐dependent roles—facilitating transcription under acute oxidative stress yet promoting resolution through antioxidant programs during recovery [70].

Beyond NF‐κB, a set of context‐dependent transcriptional programs either amplify or restrain priming. On the activating side, sterol‐responsive SREBP2 together with its escort SCAP couples cholesterol homeostasis to inflammasome competence, while NFAT5, a tonicity/stress‐responsive factor, links osmotic and inflammatory cues; both have been reported to elevate NLRP3 expression and/or increase its routing/assembly capacity in defined cell types [71, 72]. On the restraining side, the circadian repressor REV‐ERBα/NR1D1 attenuates NLRP3 transcription directly and through secondary anti‐inflammatory programs; aryl hydrocarbon receptor (AhR) ligands diminish NLRP3 expression and activation; the myeloid transcriptional repressor GFI1 curbs pro‐inflammatory gene sets; and lineage‐determinant factors IRF4/IRF8 in dendritic cells can limit inflammasome‐related transcripts in a subset‐ and context‐dependent manner (with IRF8 exerting opposite, pro‐NLRP3 effects in macrophages) [73, 74, 75, 76]. Together, these positive and negative nodes provide a mechanistic basis for why priming thresholds vary across tissues, stimuli, and circadian time.

Epigenetic modifications provide stable yet reversible control over NLRP3 expression [77]. DNA methylation of CpG islands within the NLRP3 promoter silences gene expression, with demethylation leading to transcriptional activation during inflammatory responses [78]. Histone modifications define active chromatin regions permissive for NLRP3 transcription. The enrichment of the active enhancer mark H3K27ac at the NLRP3 promoter region is positively correlated with its transcription level [79]. Moreover, histone methyltransferases such as WDR5 and DOT1L regulate NLRP3 expression through H3K4 and H3K79 methylation [80]. Post‐transcriptionally, microRNAs (miRNAs), such as miR‐223, miR‐30e‐3p, regulate NLRP3 or IL‐1β transcripts, providing rapid and reversible dampening of inflammasome potential in various disease contexts [81, 82, 83].

### 3.2 PTM Gating of Activation

Once the substrate pool exists, PTMs act as near‐instant gatekeepers of assembly and enzymatic output [84]. Ubiquitination serves as a central regulatory mechanism, with distinct ubiquitin linkages determining NLRP3 fate and function [85]. The E3 ubiquitin ligase FBXL2 targets NLRP3 for polyubiquitination and proteasomal degradation, maintaining low basal levels of NLRP3 in resting cells [86, 87]. Conversely, TRIM31 promotes K48‐linked polyubiquitination of NLRP3, which facilitates its proteasomal degradation and serves as a negative feedback mechanism [88]. The deubiquitinase BRCC3 removes ubiquitin chains from NLRP3, stabilizing the protein and enabling inflammasome activation [89]. Additionally, the linear ubiquitin assembly complex generates M1‐linked ubiquitin chains on NLRP3, which are essential for optimal inflammasome activation [90].

Phosphorylation provides another critical layer of NLRP3 regulation, with multiple kinases targeting specific residues, similarly exerts bidirectional control [91]. JNK1‐mediated phosphorylation of NLRP3 at Ser194 during the priming phase facilitates deubiquitination and protein stabilization, whereas protein kinase A phosphorylation at Ser291 induces inhibitory ubiquitination [11, 92]. Protein kinase D (PKD) phosphorylates NLRP3 at the Golgi apparatus, promoting its recruitment to the TGN dispersal site, while tyrosine phosphatases such as PTPN22 provide additional modulatory input [93, 94].

Emerging modifications, including SUMOylation, acetylation, and lactylation, further fine‐tune inflammasome activity: SUMO2/3 modification prevents NLRP3 oligomerization, reversible by SENP6/7 desumoylases [95, 96], lysine acetylation at K24 is required for full NLRP3 inflammasome activation, highlighting the importance of histone deacetylases (HDACs) in regulation [97]; Recent studies highlight the role of protein lactylation in inflammasome regulation. For instance, lactylation of citrate synthase was shown to promote NLRP3 activation in a kidney injury–fibrosis model, and lactate‐induced H3K18 lactylation upregulates ALKBH5, thereby modulating the NLRP3 pathway in macrophages [44, 45]. These findings tentatively link elevated glycolytic flux and lactylation to structural or functional modulation of NLRP3 regulation.

### 3.3 Spatial and Proteostatic Control

Beyond canonical signaling cues, the activation competence of the NLRP3 inflammasome is dictated by its spatial itinerary and proteostatic state. In resting cells, NLRP3 resides predominantly in the cytosol or associates with the endoplasmic reticulum (ER), where it is maintained in a “poised but restrained” state by the molecular chaperone complex HSP90–SGT1 [98]. This chaperone buffering preserves the protein's structural integrity and readiness for activation while preventing spontaneous oligomerization. Upon stimulation, however, NLRP3 undergoes a carefully orchestrated trafficking process that integrates subcellular localization, lipid microdomain dynamics, and PTMs, collectively shaping where and when inflammasome assembly occurs.

The ER–mitochondria‐associated membrane (MAM) axis serves as an early platform facilitating proximity between NLRP3 and its licensing partner NEK7, converting a mobile primed pool into an oligomerization‐competent species [99, 100, 101]. Concurrently, activation triggers promote the disassembly of the TGN, generating phosphatidylinositol‐4‐phosphate (PI4P)‐enriched dTGN membranes. A polybasic region within NLRP3 directly binds PI4P, creating an early supramolecular clustering hub for NLRP3 recruitment and self‐association [23]. Subsequent phosphorylation of NLRP3 by PKD and IKKβ reinforces its residency on PI4P‐rich membranes, stabilizing nucleation sites independent of potassium efflux [24].

In addition to Golgi‐associated platforms, endosomal lipid remodeling can generate alternative compartments favorable for NLRP3–ASC engagement, even in the absence of lysosomal rupture [25]. Spatial routing further extends to the cytoskeletal network: HDAC6‐mediated aggresome‐like transport directs NLRP3–ASC complexes along microtubules toward the centrosome, a process tuned by PLK1, thereby optimizing the geometric efficiency of inflammasome assembly [102]. These sequential localization steps reflect a “licensing itinerary,” in which distinct organelles provide transient docking stations that progressively prime NLRP3 for higher‐order assembly.

Parallel to spatial routing, proteostatic systems preserve the assembly competence of NLRP3 during transit. Non‐degradative chaperones such as HSP90 not only maintain proper folding but also shield exposed hydrophobic interfaces, preventing premature oligomerization. Upon activation, localization‐sensitive PTMs (e.g., S‐palmitoylation at Cys837 or phosphorylation at specific serine residues) fine‐tune NLRP3's interaction with membranes and adaptors, regulating whether the molecule remains on permissive membranes or is withdrawn for degradation [103]. While degradative pathways such as macroautophagy, chaperone‐mediated autophagy, and PINK1–Parkin–mediated mitophagy (see Section 3.4) eliminate damaged or overactivated inflammasome components, this non‐degradative proteostatic buffering ensures that only a competent subset of NLRP3 molecules is available for assembly at any given time. Mechanistically, this model explains why identical upstream signals can produce divergent inflammatory outputs across cell types and stimuli—only when spatial routing delivers NLRP3 to permissive membranes and proteostatic chaperones preserve an activation‐competent pool can ASC nucleation and caspase‐1 activation proceed efficiently.

### 3.4 Proteostasis Control: Autophagy and Mitophagy Brakes

Degradative quality‐control pathways furnish dominant brakes on inflammasome output [5]. Macroautophagy and chaperone‐mediated autophagy remove activation‐competent complexes and dampen feed‐forward amplification; defective autophagy allows accumulation of dysfunctional organelles and protein aggregates that seed activation [104]. The autophagy receptor SQSTM1/p62 binds to NLRP3 and targets it for autophagic degradation, preventing excessive inflammasome assembly [105]. PINK1‐Parkin‐mediated mitophagy removes damaged mitochondria that would otherwise release activating signals such as mitochondrial DNA and ROS [106]. Pharmacological enhancement of autophagy through mTOR inhibition or AMPK activation effectively suppresses NLRP3 inflammasome activity [107].

Beyond degradative proteostasis, endogenous inhibitors and metabolic rheostats provide additional system‐level checks [108]. Pyrin‐only proteins (POP1/POP2) compete with ASC for binding to NLRP3, preventing inflammasome assembly [109], while CARD‐only proteins (CARD16/17/18) sequester ASC and limit caspase‐1 activation [110]. However, the physiological significance of these inhibitory proteins remains unclear, as they are not expressed in mice [111]. Dopamine functions as an endogenous NLRP3 inhibitor by activating dopamine D1‐like receptors and increasing intracellular cAMP levels [112]. As mentioned in 2.2, cellular metabolism, including β‐hydroxybutyratea and omega‐3 fatty acids, profoundly influences NLRP3 inflammasome regulation through multiple interconnected pathways [37, 39, 42, 113, 114].

In sum, PTM gating, transcriptional/epigenetic priming, spatial logistics with chaperone support, and proteostatic brakes—including autophagy/mitophagy and endogenous metabolic circuits—together establish NLRP3 as a finely tuned sensor of danger and homeostasis. Their interconnectivity provides multiple checkpoints to prevent aberrant inflammation while maintaining rapid responsiveness [115, 116].

## 4 Disease Contexts and Progress in NLRP3 Inflammasome Inhibitors

The multilayered regulation of NLRP3—from transcriptional priming and post‐translational switches to organelle integrity and metabolic checkpoints—creates diverse pharmacological entry points. At the same time, aberrant NLRP3 activation contributes to a spectrum of clinically distinct disorders, in which the dominant pathogenic nodes and inflammatory milieu differ markedly. In this section, we first outline major NLRP3‐driven disease contexts that provide the clinical rationale for targeting this pathway (Section 4.1). We then discuss discovery paradigms, mechanistic nodes, and representative inhibitor lineages (Sections 4.2–4.4), which together define how pharmacology can be tailored to these disease settings. These nodes define distinct therapeutic strategies: direct blockade of NLRP3 activation and oligomerization, interference with essential protein–protein interactions, inhibition of downstream effectors, cytokine or receptor blockade, indirect modulation of upstream pathways, and targeted degradation of inflammasome components. Collectively, these strategies have generated a diverse spectrum of small molecules and biologics, ranging from early chemical probes to approved therapies (Table S1).

### 4.1 NLRP3‐Driven Disease Contexts and Rationale for Tailored Inhibition

Building on the structural insights and representative inhibitors described above, it becomes clear that the therapeutic potential of NLRP3 inhibitors is highly disease‐context dependent. Different indications are characterized by distinct combinations of genetic lesions, metabolic rewiring, organ‐specific microenvironments, and upstream triggers. Below, we summarize four representative disease clusters in which NLRP3 plays a central pathogenic role and highlight how these mechanisms motivate tailored inhibitory strategies (Table 1).

**TABLE 1:** Therapeutics targeting NLRP3 inflammasome signaling in clinical trials.

Therapeutic	Mechanism	Indication	Trial registry	Phase	Enrollment	Key outcome	Status
**IL‐1 pathway blockade(IL‐1β/IL‐1Ra/IL‐1 Trap)**							
Canakinumab (Novartis)	IL‐1β neutralizing monoclonal antibody	CAPS	NCT01105507	I	—	Confirmed safety and efficacy in CAPS patients	Completed
Canakinumab (Novartis)	IL‐1β neutralizing monoclonal antibody	CAPS	NCT01276522	III	∼60	Sustained efficacy in Schnitzler's syndrome	Approved
Canakinumab (Novartis)	IL‐1β neutralizing monoclonal antibody	FCAS	NCT01302860	II/III	—	Safety and efficacy confirmed	Completed
Canakinumab (Novartis)	IL‐1β neutralizing monoclonal antibody	FCAS	NCT00991146	I/II	—	Safety and efficacy confirmed	Completed
Canakinumab (Novartis)	IL‐1β neutralizing monoclonal antibody	FCAS	NCT00685373	II	—	Safety and efficacy confirmed	Completed
Canakinumab (Novartis)	IL‐1β neutralizing monoclonal antibody	FCAS	NCT00487708	I	—	Safety and efficacy confirmed	Completed
Canakinumab (Novartis)	IL‐1β neutralizing monoclonal antibody	AS	NCT01327846	II	10061	Lower rate of recurrent cardiovascular events independent of lipid‐lowering	Completed
Rilonacept (Regeneron)	Soluble IL‐1 decoy receptor (IL‐1 Trap)	CAPS	NCT00288704	I	—	Confirmed efficacy and safety	Completed
Rilonacept (Regeneron)	Soluble IL‐1 decoy receptor (IL‐1 Trap)	Gout	NCT02171416	II	—	Confirmed efficacy and safety	Completed
Rilonacept (Regeneron)	Soluble IL‐1 decoy receptor (IL‐1 Trap)	CAPS	NCT01045772	I/II	—	Confirmed efficacy and safety	Completed
Anakinra/HL2351 (HanAll)	Recombinant IL‐1 receptor antagonist (IL‐1Ra)	CAPS	NCT02853084	I	8	Confirmed safety and efficacy	Active
Anakinra/HL2351 (HanAll)	Recombinant IL‐1 receptor antagonist (IL‐1Ra)	Myocardial infarction	NCT04443881	I/II	8	Confirmed safety and efficacy	Active
Anakinra/HL2351 (HanAll)	Recombinant IL‐1 receptor antagonist (IL‐1Ra)	Gout	NCT01175018	I	8	Confirmed safety and efficacy	Active
Anakinra/HL2351 (HanAll)	Recombinant IL‐1 receptor antagonist (IL‐1Ra)	COVID‐19 pneumonia	NCT02578394	I/II	8	Confirmed safety and efficacy	Active
**Direct NLRP3 inflammasome inhibitors**							
Therapeutic	Mechanism	Indication	Trial registry	Phase	Enrollment	Key outcomes/Notes	Status
MCC950 (Cayman Chemical)	Small‐molecule NLRP3 inhibitor	Rheumatoid arthritis	NCT02953709	II	∼200	Terminated due to hepatotoxicity	Terminated
DFV890/IFM‐2427 (IFM Therapeutics)	Small‐molecule NLRP3 inhibitor	FCAS	NCT04269469	I/II	100	First‐in‐human study initiated	Active
DFV890/IFM‐2427 (IFM Therapeutics)	Small‐molecule NLRP3 inhibitor	FCAS	NCT04868968	II	4	To assess the safety and efficacy	Active
DFV890/IFM‐2427 (IFM Therapeutics)	Small‐molecule NLRP3 inhibitor	Osteoarthritis	NCT04886258	II	108	To assess the safety and efficacy	Active
DFV890/IFM‐2427 (IFM Therapeutics)	Small‐molecule NLRP3 inhibitor	Myeloid diseases	NCT05552469	1b	80	To assess the safety and efficacy	Active
DFV890/IFM‐2427 (IFM Therapeutics)	Small‐molecule NLRP3 inhibitor	COVID‐19 pneumonia	NCT04382053	II	143	Confirmed efficacy and safety for SARS‐CoV‐2‐induced pneumonia	Active
Inzomelid (Roche)	NLRP3 inhibitor	Healthy subjects	NCT04015076	I	94	Evaluate safety and PK/PD	Active
[] (Roche)	NLRP3 inhibitor	CAPS	NCT04015076	I	94	Evaluate safety and PK/PD	Active
Inzomelid (Roche)	NLRP3 inhibitor	Parkinson's disease	NCT04338997	I	—	Withdrawn	Withdrawn
Inzomelid (Roche)	NLRP3 inhibitor	Knee OA	EUCTR2020‐006104‐17	II	108	Study efficacy, safety, and tolerability	Active
Inzomelid (Roche)	NLRP3 inhibitor	FCAS	EUCTR2020‐005948‐33	II	6	Assess safety and efficacy	Active
RO7486967/Somalix (Roche)	NLRP3 inhibitor	CAPS	NCT04086602	I	64	Assess safety and PK/PD	Active
RO7486967/Somalix (Roche)	NLRP3 inhibitor	Coronary artery disease	EudraCT 2020‐000942‐32	IIb	132	Evaluate safety and efficacy to reduce CRP	Active
RO7486967/Somalix (Roche)	NLRP3 inhibitor	Parkinson's disease	ISRCTN85338453/NCT05924243	Ib	72	Assess safety and PK/PD	Active
RO7486967/Somalix (Roche)	NLRP3 inhibitor	COPD	ISRCTN17672960	I	106	Assess safety and PK/PD	Active
RO7486967/Somalix (Roche)	NLRP3 inhibitor	Ulcerative colitis	ISRCTN16847938	Ib	19	Phase 1b study evaluating safety, PK/PD	Active
NT‐0796 (NodThera)	CNS‐penetrant NLRP3 inhibitor	Healthy volunteers	ACTRN12621001082897	I	88	Demonstrated safety	Active
NT‐0249 (NodThera)	CNS‐penetrant NLRP3 inhibitor	Healthy volunteers	ACTRN12622000195752	I	72	Assess safety and PK/PD	Active
NT‐0167 (NodThera)	Oral NLRP3 inhibitor	Healthy volunteers	ACTRN12620000685910	I	80	Assess safety and PK/PD	Active
ZYIL1 (Zydus Lifesciences)	Oral NLRP3 inhibitor	Healthy volunteers	NCT04972188/NCT04731324	I	18	Confirmed safety and PK/PD	Completed
ZYIL1 (Zydus Lifesciences)	Oral NLRP3 inhibitor	CAPS	NCT05186051	IIa	3	Incidence and severity of adverse events	Completed
Dapansutrile (OLT1177, Olatec)	Oral NLRP3 inhibitor	Healthy volunteers	NCT02134964	I	36	Confirmed safety and PK/PD	Completed
Dapansutrile (OLT1177, Olatec)	Oral NLRP3 inhibitor	Systolic heart failure	NCT03534297	Ib	30	Confirmed safety and PD	Completed
Dapansutrile (OLT1177, Olatec)	Oral NLRP3 inhibitor	Myocardial infarction	NCT05880355	I	60	Evaluate efficacy	Active
Dapansutrile (OLT1177, Olatec)	Oral NLRP3 inhibitor	PD‐1 refractory advanced melanoma	NCT04971499	I/II	26	Assess efficacy	Active
Dapansutrile (OLT1177, Olatec)	Oral NLRP3 inhibitor	Schnitzler's syndrome	NCT03595371/EUCTR2017‐003282‐98‐NL	II	10	Assess safety and efficacy	Active
Dapansutrile (OLT1177, Olatec)	Oral NLRP3 inhibitor	COVID‐19‐induced early cytokine release syndrome	NCT04540120	II	49	Terminated due to changing pandemic conditions	Terminated
Dapansutrile (OLT1177, Olatec)	Oral NLRP3 inhibitor	Acute gout flare	NCT05658575	II/III	300	Evaluate efficacy in reducing joint pain	Active
Tranilast (Kissei)	Oral NLRP3 inhibitor	CAPS	NCT03923140	II	71	Observe efficacy and safety	Active
Tranilast (Kissei)	Oral NLRP3 inhibitor	COVID‐19	IRCT20200419047128N1	II/III	60	Confirmed effectiveness as adjuvant therapy	Active
VTX‐2735 (Ventyx Biosciences)	Oral NLRP3 inhibitor	CAPS	NCT05812781	II	10	Evaluate safety and effectiveness	Active
VTX‐3232 (Ventyx Biosciences)	CNS‐penetrant NLRP3 inhibitor	Healthy volunteers	—	I	100	Evaluate safety and effectiveness	Active
VENT‐02	Small‐molecule NLRP3 inhibitor	Parkinson's disease	NCT05276895	I	∼100	Brain‐penetrant; completed Phase I in healthy volunteers	Active
VENT‐02	Small‐molecule NLRP3 inhibitor	Alzheimer's disease	NCT05276895	I	∼100	Brain‐penetrant; completed Phase I in healthy volunteers	Active
YQ128	Small‐molecule NLRP3 inhibitor	Alzheimer's disease	—	I	∼100	Brain‐penetrant; anti‐inflammatory; Phase I in healthy volunteers	Active
YQ128	Small‐molecule NLRP3 inhibitor	Traumatic brain injury	—	I	∼100	Brain‐penetrant; anti‐inflammatory; Phase I in healthy volunteers	Active
JT002	Small‐molecule NLRP3 inhibitor	Healthy volunteers	—	I	∼100	Inhibits NLRP3‐dependent cytokine production	Active
GDC‐2394	Small‐molecule NLRP3 inhibitor	Healthy volunteers	NCT05552469	I	∼100	Safety, PK/PD evaluation	Active
**Upstream/alternative inflammasome modulators**							
Therapeutic	Mechanism	Indication	Trial registry	Phase	Enrollment	Key outcomes/Notes	Status
HT‐6184 (Halia Therapeutics)	NEK7 kinase inhibitor	Chronic inflammation‐driven diseases	NCT05447546	I	32	Evaluate safety and PK/PD	Active
Colchicine (Generic)	Microtubule inhibitor	Cardiovascular disease	NCT02551094/ACTRN12610000293066/ACTRN12614000093684	II–III	2023.6	FDA approved for CAD	Approved
Colchicine (Generic)	Microtubule inhibitor	Myocardial reperfusion injury	NCT05734612	III	80	Evaluate the effect on myocardial reperfusion injury	Active
Colchicine (Generic)	Microtubule inhibitor	COVID‐19	NCT04326790/RBR‐8jyhxh	II	180	Inhibited NLRP3 inflammasome activation	Active
Colchicine/Tranilast/Oridonin	NLRP3 inhibitor	CAD after PCI	NCT05130892	IV	132	Evaluate the efficacy of different NLRP3 inhibitors	Active
Quercetin + Fisetin	Natural flavonoids/NLRP3 suppression	OA	NCT05276895	—	60	Determine efficacy in reducing knee symptoms and effusion‐synovitis	Active
ADS032 (Adis Insight)	Dual NLRP3 & NLRP1 inflammasome inhibitor	Interstitial lung disease	ISRCTN35867933	0	50	Assess efficacy	Active
Nibrozetone (RRX‐001)	Epigenetic/nitrosylating agent, NLRP3 inhibition + Nrf2 activation	Cancers	NCT02215512/NCT02452970/NCT02518958/NCT01359982	I–III	—	Confirmed safety and efficacy	Active
Pralnacasan (Idun)	Caspase‐1 inhibitor	RA	NCT04269469	II	100	Discontinued	Discontinued
Pralnacasan (Idun)	Caspase‐1 inhibitor	OA	NCT04269469	II	100	Discontinued	Discontinued
Belnacasan (VX‐765)	Caspase‐1 inhibitor	Epilepsy	NCT01048255/NCT01501383/NCT00205465	II	60	Evaluate efficacy in treatment‐resistant patients	Active
Belnacasan (VX‐765)	Caspase‐1 inhibitor	Psoriasis	NCT01048255/NCT01501383/NCT00205465	II	64	Evaluate efficacy	Active
Belnacasan (VX‐765)	Caspase‐1 inhibitor	COVID‐19	NCT05164120	II	43	Assess efficacy	Active
Fenofibrate	PPAR‐α agonist	IBD	NCT05781698	II	60	Repurposing fenofibrate to modulate mTOR/NLRP3 inflammasome	Active
Melatonin	Immunomodulator	COVID‐19	NCT04409522	—	55	Evaluate therapeutic effects via NLRP3 inhibition	Active
AC‐203	Inflammasome/IL‐1β pathway modulator	Inherited epidermolysis bullosa	NCT03468322	II	9	Test efficacy and safety	Active
AC‐203	Inflammasome/IL‐1β pathway modulator	Bullous Pemphigoid	NCT03286582	II	10	Terminated with partial enrollment	Terminated
HY209	GPCR19 agonist	Atopic dermatitis	NCT03492398	I	56	Assess GPCR19 agonist inhibiting NLRP3 inflammasome	Active
HY209	GPCR19 agonist	Atopic dermatitis	NCT04530643	II	80	Assess GPCR19 agonist inhibiting NLRP3 inflammasome	Active

Data sources: Public clinical trial registries and regulatory databases (e.g., ClinicalTrials.gov; EU Clinical Trials Register/EudraCT; Chinese Clinical Trial Registry/ChiCTR; FDA; NMPA; PMDA publicly available registration records), accessed November 2025.

Abbreviations: AD, Alzheimer's disease; AS, atherosclerosis; CAD, coronary artery disease; CAPS, Cryopyrin‐associated periodic syndrome; COPD, chronic obstructive pulmonary disease; COVID‐19, coronavirus disease 2019; FCAS, familial cold autoinflammatory syndrome; GPCR, G protein–coupled receptor; IBD, inflammatory bowel disease; IL‐1Ra, interleukin‐1 receptor antagonist; MAD, multiple ascending dose; MI, myocardial infarction; NEK7, NIMA‐related kinase 7; NLRP3, nucleotide‐binding domain, leucine‐rich–containing family, pyrin domain–containing‐3; Nrf2, nuclear factor erythroid 2–related factor 2; OA, osteoarthritis; PD (in pharmacology), pharmacodynamics; PD, Parkinson's disease; PK, pharmacokinetics; PPAR‐α, peroxisome proliferator–activated receptor alpha; RA, rheumatoid arthritis; RCT, randomized controlled trial; SAD, single ascending dose; UC, ulcerative colitis.

#### 4.1.1 Autoinflammatory Diseases

CAPS exemplifies the genetic contribution to NLRP3‐driven diseases. Mutations in NLRP3 lead to constitutive inflammasome activation and excessive IL‐1β secretion, manifesting as familial cold autoinflammatory syndrome (FCAS), Muckle‐Wells syndrome (MWS), and neonatal‐onset multisystem inflammatory disease (NOMID) [117]. Functional categorization of CAPS‐associated mutations into five classes has provided a framework for linking genetic variation to differential cytokine release and cell death pathways, offering a rationale for patient‐specific interventions. For instance, the newly identified pSer595Asn mutation causes hearing loss via enhanced inflammasome activity, yet anti‐IL‐1 therapy has successfully reversed this phenotype in pediatric patients [118]. Mutations such as Q703K and A350V, widely documented in Infevers and Eurofever databases, similarly illustrate the spectrum of NLRP3 activation in CAPS [119, 120, 121]. Beyond mutation‐driven activation, sustained inflammation involves metabolic reprogramming and deubiquitination pathways, while the HSP90β–SGT1 chaperone complex and Ca^2^^+^‐dependent mechanotransduction through KCNN4 and PIEZO channels further amplify inflammasome activity [122, 123, 124]. These mechanistic insights not only explain variable disease severity across patients but also highlight therapeutic vulnerabilities, such as HSP90β inhibition or blockade of KCNN4, that could complement existing IL‐1–directed therapies. Indeed, IL‐1 inhibitors, including anakinra, canakinumab, and rilonacept, remain the clinical standard, though responses vary depending on disease severity and mutation spectrum [125, 126, 127, 128, 129, 130]. Notably, some CAPS mutations confer resistance to MCC950 but retain responsiveness to alternatives like CY‐09, underscoring the promise of genotype‐guided “tailored inhibition” [131].

Recent studies have further clarified genotype‐dependent responses to NLRP3 inhibition. For example, specific NLRP3 gain‐of‐function mutations not only dictate disease severity but also influence inhibitor efficacy, suggesting that genetic profiling could guide patient‐specific therapy [131]. Moreover, small molecule inhibitors such as MCC950 have demonstrated robust suppression of IL‐1β release in patient‐derived cells, although dose‐limiting hepatotoxicity highlights the need for safer derivatives [132]. Emerging PROTAC‐based NLRP3 degraders have shown potent inflammasome suppression in preclinical models, providing a promising avenue for future precision therapy [133].

#### 4.1.2 Inflammatory Arthritis

Rheumatoid arthritis (RA), osteoarthritis (OA), and gouty arthritis (GA) highlight the interplay of innate immunity, crystal‐induced activation, and inflammasome signaling in joint inflammation. In RA, excessive T and B cell activation converges on NLRP3‐mediated IL‐1β release, with genetic variants in NLRP3 and its regulators (CARD8, A20) influencing susceptibility and disease severity [134, 135, 136]. In gout, monosodium urate (MSU) and calcium pyrophosphate crystals activate macrophage NLRP3, driving pyroptosis and amplifying neutrophil infiltration [137]. In OA, where mechanical stress and cartilage degradation dominate, uric acid and other DAMPs act as triggers for NLRP3 activation, accelerating cartilage loss and synovitis [138, 139, 140, 141]. Consistent with these mechanistic insights, therapeutic inhibition of IL‐1β alleviates gout symptoms, while small‐molecule inhibitors such as MCC950 and OLT1177 show efficacy in preclinical arthritis models [142, 143]. Ketogenic diets, β‐hydroxybutyrate, and certain endogenous metabolites have also been shown to attenuate NLRP3 activation [144, 145]. Ongoing clinical trials with Dapansutrile are expected to clarify the potential of targeting NLRP3 across diverse arthritis phenotypes [146]. Collectively, these studies suggest that tailoring inhibition to pathogenic nodes—whether pyroptosis in gout, ASC signaling in RA, or calcification‐associated triggers in OA—could provide disease‐specific benefit.

In RA and other sterile inflammatory arthritis models, recent evidence highlights ASC's role beyond canonical NLRP3 activation. ASC‐deficient CD4^+^ T cells have increased IL‐10 production, impairing bystander T‐cell proliferation, suggesting that inflammasome‐independent functions of ASC may modulate local inflammation [147, 148]. Consequently, NLRP3 inhibition strategies may require careful consideration of ASC‐dependent pathways to maximize therapeutic benefit. Additionally, the development of covalent inhibitors such as oridonin derivatives shows promise in selectively blocking NLRP3 assembly without affecting basal immune function [149, 150].

#### 4.1.3 Inflammatory Bowel Disease

Both Crohn's disease (CD) and ulcerative colitis (UC) exhibit strong genetic and immunological links to NLRP3 dysregulation. Variants such as NLRP3 R779C or CARD8 T60 promote persistent inflammasome activation, correlating with disease severity and resistance to anti‐TNF‐α therapy [151, 152]. Molecular mechanisms, including BTK‐mediated phosphorylation, OTUD6A‐ and BRCC3‐mediated deubiquitination, and METTL3‐driven m6A modification, amplify inflammasome activity and pyroptosis [153, 154]. Conversely, protective mechanisms such as IRGM‐driven autophagic degradation of NLRP3, HSP‐mediated suppression, or IL‐10–dependent mitophagy maintain mucosal balance [155, 156]. The gut microbiota further modulates NLRP3 responses; for example, Bacteroides fragilis and Lactobacillus acidophilus alleviate colitis via SCFA production and mitophagy, whereas dysbiosis promotes pathogenic signaling [157, 158]. Importantly, NLRP3 not only drives intestinal inflammation but also contributes to extraintestinal manifestations and colitis‐associated cancer. Preclinical studies with MCC950, natural products, and FDA‐approved drugs such as glyburide demonstrate clear benefits, though early‐phase clinical trials with compounds like selnoflast have yielded mixed results [159, 160, 161]. These findings emphasize that therapeutic success will likely depend on precise targeting of patient‐specific pathways—whether genetic variants, microbiota composition, or metabolic context—to achieve durable disease control.

Recent preclinical work has introduced autophagy‐tethering compounds that degrade NLRP3, demonstrating potent anti‐inflammatory effects in DSS‐induced colitis models. For instance, compound MC‐ND‐18 achieved marked suppression of colonic IL‐1β and histological inflammation, illustrating the potential of combining inflammasome inhibition with cellular degradation pathways [162]. These findings suggest that patient stratification based on NLRP3 activity and autophagy status could improve therapeutic precision in inflammatory bowel disease (IBD).

#### 4.1.4 Cardiovascular Diseases: Atherosclerosis and Myocardial Infarction

The contribution of NLRP3 to atherosclerosis and myocardial infarction highlights its role as a driver of sterile inflammation in cardiovascular pathology. Cholesterol crystals, oxidized LDL, and hemodynamic stress activate NLRP3, promoting endothelial dysfunction, plaque progression, and thrombosis [163, 164]. Similarly, ischemia/reperfusion injury after myocardial infarction (MI) triggers mitochondrial cfDNA/TLR9 signaling, activating NLRP3 and exacerbating tissue injury [165]. Inhibiting NLRP3 reduces infarct size and improves remodeling, as shown in animal models treated with MCC950 or VX‐765. Clinical translation is progressing: Dapansutrile has entered Phase I trials in systolic heart failure and MI, while colchicine—an established anti‐inflammatory agent now recognized as an NLRP3 inhibitor—has gained FDA approval for cardiovascular indications [166]. Additional approaches, such as bifunctional nanoparticles targeting macrophages or agents blocking Txnip/NLRP3 signaling, demonstrate the potential of tailoring inhibition to the cardiovascular context [167, 168]. Importantly, genetic backgrounds—such as somatic mutations like TET2 mutations driving clonal hematopoiesis—may further refine therapeutic decision‐making [169].

In cardiovascular diseases such as atherosclerosis and myocardial infarction, NLRP3 activation is influenced by metabolic stress and macrophage phenotype. Recent studies highlight that inhibitors like OLT1177 can reduce myocardial inflammation and improve cardiac function in preclinical MI models, while avoiding systemic immunosuppression [170]. Additionally, drug repurposing approaches with tranilast have been demonstrated anti‐atherosclerotic effects by blocking NLRP3 oligomerization [171]. These findings underscore the importance of tailoring NLRP3‐targeted therapy according to disease‐specific inflammatory triggers and patient metabolic profiles.

Taken together, across diseases ranging from rare hereditary syndromes to highly prevalent cardiovascular disorders, the application of NLRP3 inhibition demonstrates that a “one‐size‐fits‐all” strategy is unlikely to succeed. Instead, the integration of genetic, molecular, and environmental determinants of NLRP3 activation provides a path toward precision therapies that match the heterogeneity of inflammasome‐driven diseases. Collectively, these findings highlight that “one‐size‐fits‐all” NLRP3 inhibition is unlikely to achieve optimal efficacy. Instead, integrating disease‐specific mechanisms, patient genetics, and drug pharmacology will be critical for developing precision‐tailored therapies. Future efforts combining small molecule inhibitors, targeted degraders, and genotype‐guided approaches are expected to advance the clinical translation of NLRP3‐targeted interventions across multiple inflammatory conditions.

### 4.2 Discovery Paradigms and Translational Milestones

**FIGURE 5:**
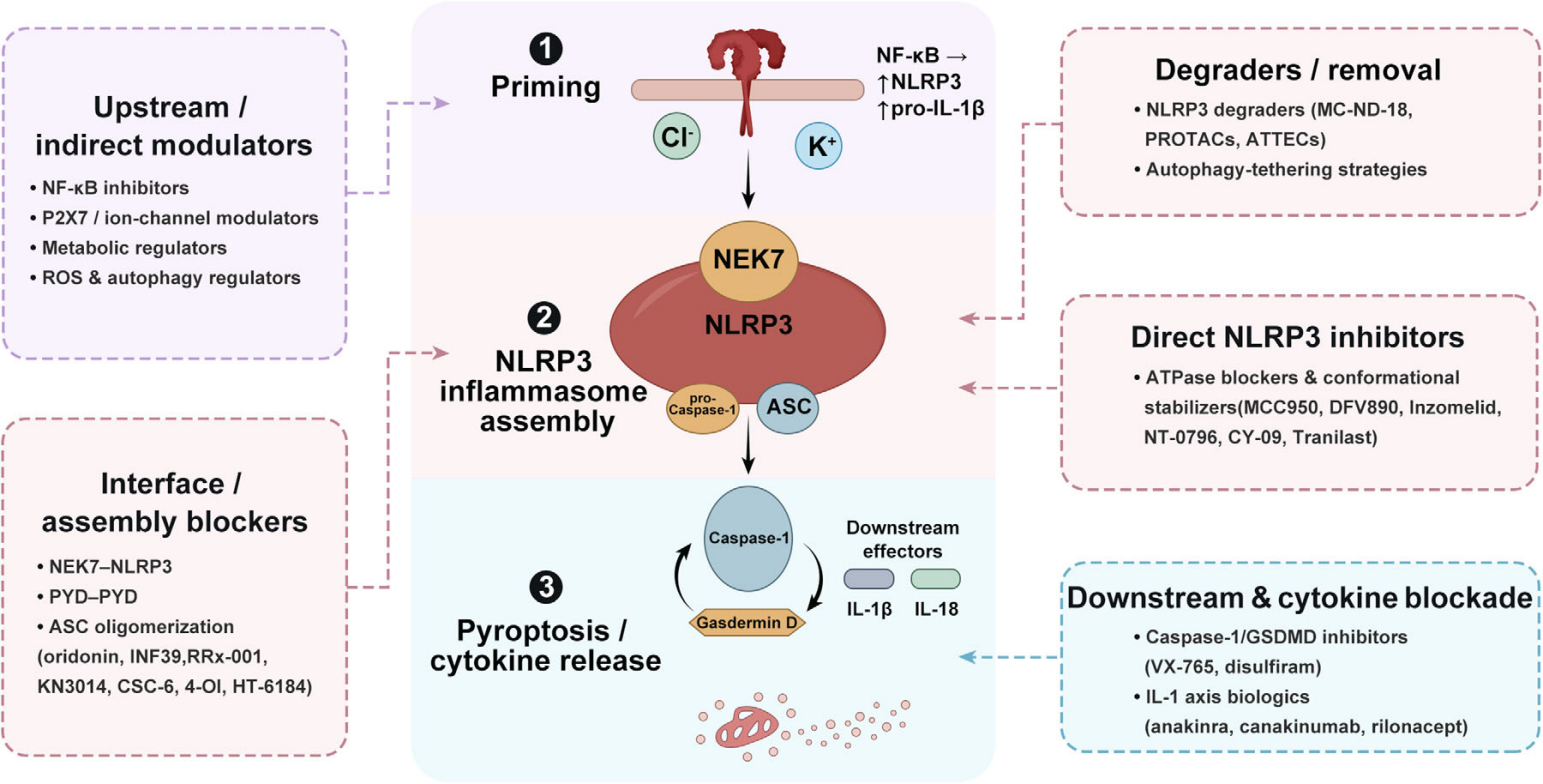
Therapeutic intervention points along the NLRP3 inflammasome cascade. Potential pharmacological strategies targeting the NLRP3 pathway at distinct stages: (1) upstream/indirect modulators that suppress priming (e.g., NF‐κB inhibition), regulate ion channels (e.g., P2×7), metabolic pathways, ROS, and autophagy; (2) interface/assembly blockers disrupting key protein–protein interactions (e.g., NEK7–NLRP3 licensing, PYD–PYD interactions, and ASC oligomerization); (3) direct NLRP3 inhibitors that block ATPase activity or stabilize inactive conformations; (4) degraders/removal approaches (e.g., NLRP3 degraders and autophagy‐tethering strategies); and (5) downstream blockade targeting caspase‐1/GSDMD or IL‐1 axis biologics to prevent cytokine release and pyroptosis. ASC, apoptosis‐associated speck‐like protein containing a CARD; ATPase, adenosine triphosphatase; ATTECs, autophagosome‐tethering compounds; GSDMD, gasdermin D; IL‐1β, interleukin‐1 beta; IL‐18, interleukin‐18; NEK7, NIMA‐related kinase 7; NF‐κB, nuclear factor kappa B; NLRP3, NOD‐like receptor family pyrin domain containing 3; P2×7, purinergic receptor P2×7; PROTACs, proteolysis‐targeting chimeras; PYD, pyrin domain; ROS, reactive oxygen species.

No single discovery paradigm has proven universally optimal for NLRP3 inhibitors; successful candidates have emerged from complementary strategies that integrate chemical diversity, biological relevance, and mechanistic insight. Below, we summarize key approaches with representative milestones, which together underpin the therapeutic strategies applied to the disease settings outlined in Section 4.1 (Figure 5).

#### 4.2.1 Phenotypic Drug Discovery

Phenotypic drug discovery (PDD) played a foundational role in revealing the druggability of NLRP3 pathways. Even before NLRP3 was identified, glyburide was shown to inhibit IL‐1β release, highlighting the feasibility of targeting this axis [172]. Subsequent cell‐based assays monitoring IL‐1β secretion, ASC speck formation, or pyroptotic cell death enabled the discovery of MCC950 (CRID3) and numerous natural products or small molecules [173]. By directly capturing functional endpoints, PDD identified compounds affecting complex inflammasome assembly, including indirect metabolic modulators. While some hits required subsequent mechanistic deconvolution, this approach provided critical chemical diversity and biological relevance for early‐stage discovery.

#### 4.2.2 Structure‐Guided and Target‐Informed Discovery

The resolution of NLRP3 structures via cryo‐EM and X‐ray crystallography (7ALV, 7VTQ, 7PZC, 8ETR, etc.) has facilitated structure‐guided discovery (Table S1) [174]. These studies revealed binding modes of MCC950, NP3‐146, and others to the NACHT domain, including interactions with Walker A/B residues and hydrophobic pockets spanning WHD, HD1, and HD2 [56, 174, 132]. Structural insights guided rational SAR modifications, improved selectivity and metabolic stability, and clarified resistance associated with CAPS mutations [132, 175]. Nonetheless, structural guidance alone cannot fully anticipate clinical toxicity, as exemplified by GDC‐2394–related hepatotoxicity [176].

#### 4.2.3 High‐Throughput and High‐Content Screening

High‐throughput (HTS) and high‐content screening (HCS) have expanded the chemical space accessible for NLRP3 inhibitors. Libraries of 10^3^–10^5^ compounds have yielded hits such as PU‐H71 and MPC‐3100 using specialized assays including FLECS, split‐luciferase, and ATP‐competition formats [173, 177, 178, 179]. These approaches enable rapid identification of ATP‐pocket binders and modulators of inflammasome assembly, though promising hits typically require extensive mechanistic and pharmacokinetic optimization.

#### 4.2.4 Computational and AI‐Driven Discovery

Computational approaches, including pharmacophore modeling, virtual libraries exceeding tens of millions of compounds, and deep learning–designed scaffolds (e.g., SN3‐1), have accelerated scaffold innovation and chemical space exploration [180, 181, 182]. While these methods can generate high‐affinity, in vivo–active inhibitors, they rely on high‐quality structural templates or predictive biomarkers and require substantial experimental validation.

The most effective discovery programs combine phenotypic and structure‐guided strategies, supported by HTS, virtual screening, and AI, while integrating early DMPK, hepatotoxicity assessment, and human‐relevant cellular assays. Early target deconvolution, structural determination, and toxicology studies have proven critical for translating NLRP3 inhibitors from cell‐based discovery to clinical development.

### 4.3 Therapeutic Nodes and Mechanistic Classification

The NLRP3–IL‐1 axis can be therapeutically modulated at multiple levels, and for clarity we group interventions into several operational classes. First, direct NLRP3 inhibitors—typically small molecules targeting the NACHT domain—block ATP hydrolysis and stabilize an inactive conformation, thereby preventing NLRP3 oligomerization and ASC nucleation. These agents intervene at the apex of the pathway and are effective against both canonical and non‐canonical triggers, though challenges include isoform selectivity, on‐target safety, and resistance mutations observed in CAPS [60, 183]. Second, interface and assembly blockers act on critical protein–protein interactions such as NEK7–NLRP3, PYD–PYD, or CARD–CARD, and encompass small molecules, peptides, macrocycles, and degrader‐based approaches; they offer high mechanistic specificity but often face druggability barriers [184, 185]. Third, downstream protease and pore inhibitors—targeting caspase‐1 or GSDMD—halt IL‐1β/IL‐18 maturation or pyroptotic pore formation, which is valuable in diseases where cytokine release or pyroptosis dominate pathology; examples include caspase‐1 inhibitors (VX‐765) and GSDMD inhibitors (disulfiram) [186, 187, 188]. Fourth, cytokine or receptor blockade is well validated clinically: biologics such as anakinra, canakinumab, and rilonacept neutralize IL‐1 signaling and provide benchmarks in clinical trials, though they do not block upstream inflammasome functions such as IL‐18 release or pyroptosis [189, 190]. Fifth, indirect and upstream modulators suppress triggers of NLRP3 activation, including extracellular ATP/P2×7 signaling, ionic flux, defective autophagy, or metabolic dysregulation; such interventions may be particularly relevant in diseases driven by metabolic or stress‐related activation [191, 192, 193]. Finally, protein degradation and targeted removal strategies—including PROTACs and autophagy‐tethering compounds—aim to eliminate NLRP3 or its critical partners, offering an attractive approach in constitutively active or mutation‐driven contexts [194, 195].

This mechanistic classification not only highlights the breadth of potential therapeutic entry points but also supports a precision‐tailored approach, in which interventions are selected based on disease‐specific pathogenic nodes, inflammatory milieu, patient genotype, and metabolic context detailed in 4.4. In the following sections, we detail each class with representative small molecules and biologics, mechanistic rationale, pharmacological profiles, safety considerations, and translational status.

### 4.4 Representative Molecular Lineages of NLRP3 Inhibitors

**FIGURE 6:**
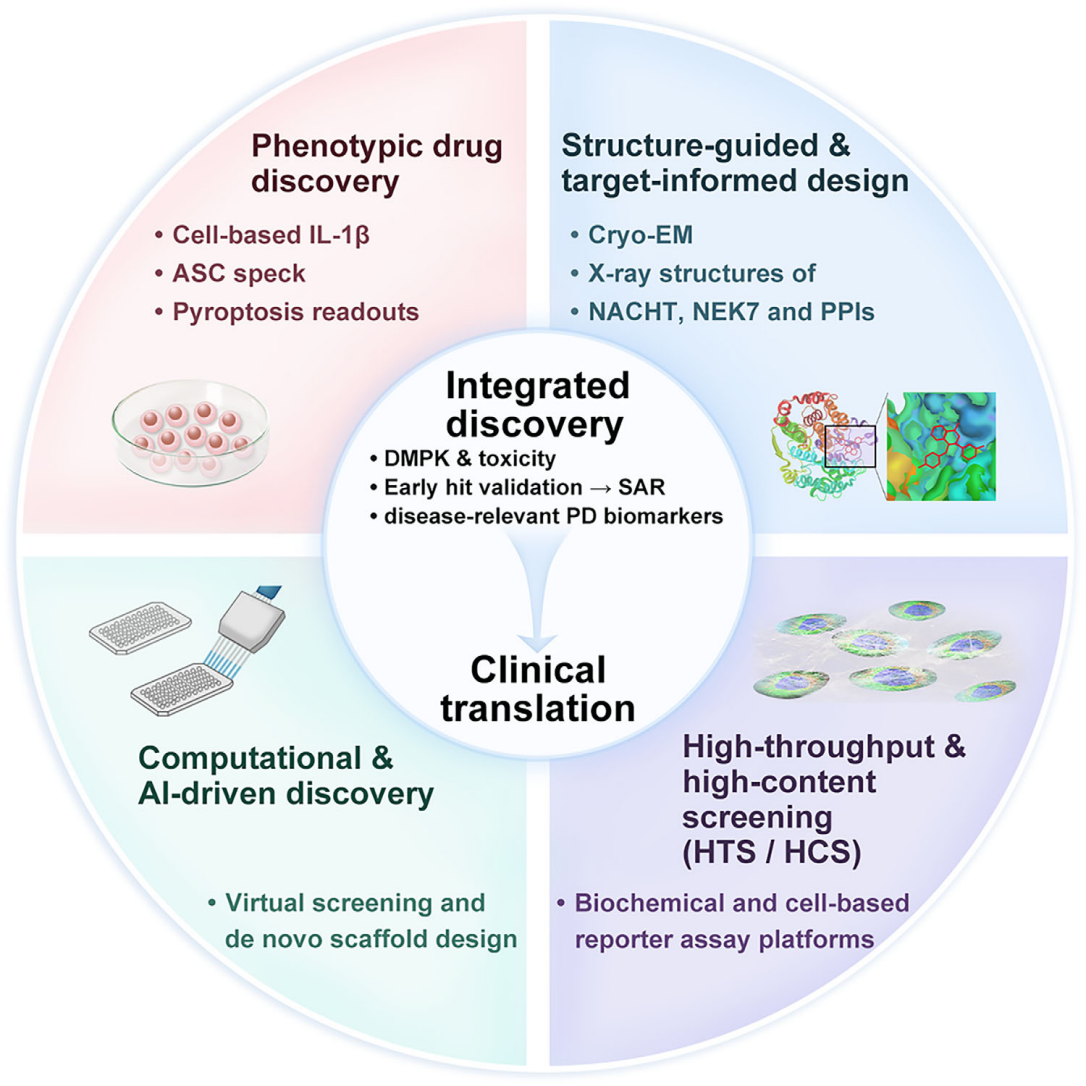
Integrated discovery framework for NLRP3‐targeted therapeutics. Conceptual workflow illustrating integrated drug discovery strategies converging on clinical translation, including phenotypic drug discovery (cell‐based IL‐1β, ASC speck, and pyroptosis readouts), structure‐guided/target‐informed design (cryo‐EM and X‐ray structures of NACHT/NEK7 and relevant PPIs), computational and AI‐driven discovery (virtual screening and de novo scaffold design), and high‐throughput/high‐content screening platforms, supported by early DMPK/toxicity evaluation, hit validation and SAR optimization, and incorporation of disease‐relevant pharmacodynamic biomarkers. ASC, apoptosis‐associated speck‐like protein containing a CARD; Cryo‐EM, cryo‐electron microscopy; DMPK, drug metabolism and pharmacokinetics; HCS, high‐content screening; HTS, high‐throughput screening; IL‐1β, interleukin‐1 beta; NACHT, NAIP/C2TA/HET‐E/TP1 domain; NEK7, NIMA‐related kinase 7; PD, pharmacodynamic; PPIs, protein–protein interactions; SAR, structure–activity relationship.

NLRP3 inflammasome inhibitors can be broadly classified according to their chemical family and mechanistic strategy. Below, we summarize representative lineages, highlighting benchmark molecules, structural insights, pharmacological optimization, translational challenges, and lessons learned for future development (Figure 6, Table 2, and a detailed summary of more than 100 compounds in Table S1).

**TABLE 2:** Representative NLRP3 inflammasome pathway modulators.

Name	Scaffold/Class	Activity	Mechanism/Binding site	Key preclinical models	Development status	Ref.
MCC950 (CP‐456,773)	Diaryl‐sulfonylurea	IC_50_ ≈ 8 nM (BMDMs), ≈7–10 nM (PBMC)	Direct NLRP3‐NACHT binder (Walker B/ATPase region); stabilizes inactive conformation	Broad: CAPS models, EAE, NASH, AD, cardiovascular models	Potent tool/advanced preclinical; clinical development halted (Phase II RA) due to liver safety signals	[183]
DFV890/IFM‐2427 (aka IFM/DFV890)	Sulfonyl/sulfonamide‐derived small molecule	nM–sub‐µM activity (cell assays)	NLRP3 NACHT/inflammasome assembly inhibitor (oral)	Animal inflammation models; tested in COVID‐19 pneumonia RCT	Phase I/ II trials; human PK/PD & early efficacy studies reported	[196]
Inzomelid (IZD174)	MCC950‐like sulfonylurea analogue (Inflazome → Roche)	nM cell activity reported	NLRP3 NACHT binder (MCC950 family targeting ATPase pocket)	CAPS, healthy volunteer PK/PD	Phase I/II trials (some trials ongoing/withdrawn per registry)	[197, 198]
Somalix/RO7486967 (selnoflast)	MCC950‐like oral small molecule	nM–sub‐µM in vitro	Oral NLRP3 inhibitor (NACHT targeting)	Arthritis, cardiovascular inflammation models	Phase I/II (several indications)	[198]
NT‐0796 (NodThera)	CNS‐penetrant small molecule (NodThera series)	sub‐nM to nM (company PBMC/animal data)	NLRP3 inhibitor (NACHT); brain penetration engineered	Neuroinflammation/PD models; healthy volunteer PK	Phase I completed	[199]
ZYIL1 (Zydus)	Oral small molecule (sulfonyl‐like)	nM–µM PK/PD readouts in Phase I	NLRP3 inhibitor (reported NACHT interaction)	Healthy volunteers; small CAPS cohorts	Phase I completed; small Phase IIa in CAPS reported	[198]
OLT1177 (dapansutrile)	β‐sulfonyl nitrile small molecule	nM activity (cell lines, J774); oral PD effects in humans	Inhibits NLRP3 activation and ASC assembly; reduces caspase‐1 activation	Gout models, knee OA, heart failure, PD studies; human Phase I/II studies show safety/PK	Multiple Phase I/II trials; Phase II/III programs ongoing for gout/other	[142, 170, 200, 201, 202]
CY‐09	Cyanopyrazole/cyano‐pyrimidine class (small molecule)	µM range in BMDMs (original report ≈2.4 µM)	Binds Walker‐A ATP‐binding motif; inhibits ATP hydrolysis	CAPS mouse models, metabolic disease models	Preclinical (tool lead); SAR efforts ongoing	[178]
GDC‐2394 (Genentech)	MCC950‐derived pyrazoloxazine scaffold	nM in vitro	NLRP3 NACHT binder (structural support); oral	Preclinical inflammasome models; entered human SAD/MAD cohorts	Phase I — halted after severe DILI cases during the DDI cohort	[175, 176]
SN3‐1	Novel tricyclic/polycyclic small molecule (AI‐designed candidate)	IC_50_ ≈ 8 nM (THP‐1); KD ≈ 6.9 nM	NACHT binding (crystallography reported); similar pocket to MCC950 family	Peritonitis, gouty arthritis, and neuro models (rodents)	Preclinical lead (advanced optimization)	[182]
NP3‐562/NP3 series	Tricyclic (non‐sulfonyl) small molecules	whole blood IC_50_ ≈ 214 nM (NP3‐562)	NACHT binding (distinct pocket vs MCC950; X‐ray support)	Peritonitis model; brain‐penetrant analogs for neurodegeneration	Preclinical (structural biology documented)	[203]
P33 (Ex‐63 optimized)	Tricyclic/diphenylamine scaffold	nM IC_50_ in THP‐1/BMDM; KD ≈17.5 nM	Binds NLRP3; blocks ASC oligomerization	Peritonitis, systemic inflammation models; favorable oral PK in animals	Preclinical candidate with good oral exposure	[204]
Oridonin (and derivatives)	Natural diterpenoid (ent‐kaurane)	µM → nM reported (derivatives)	Covalent modification of Cys279 in NACHT → disrupts NEK7 binding	Peritonitis, MSU gout, TBI, and MI models	Preclinical; multiple derivatization efforts	[205, 206]
RRx‐001 (nibrozetone)	Nitric‐oxide/epigenetic modulator; nitro‐bearing scaffold	∼200–300 nM (BMDMs) reported	Covalent modification of NLRP3 (Cys409) → blocks NEK7 interaction; Nrf2 activation	Inflammation models, tumor models; some human oncology trials	Clinical anticancer programs (multi‐indication); NLRP3 mechanism documented	[207]
INF series (INF39/INF172/INF58)	Michael‐acceptor/acrylate derivatives	µM range (THP‐1/BMDM)	Inhibit NLRP3 ATPase; some block NEK7‐NLRP3	IBD models, colitis, and peritonitis	Preclinical/optimization stage	[208, 209]
Erianin/Costunolide (natural product derivatives)	Natural product sesquiterpene/diterpenoid derivatives	reported nM–µM depending on assay	Bind NACHT cysteines (e.g., Cys463) → affect ATPase	Peritonitis, type‐2 diabetes models, and gout models	Preclinical (tool leads)	[210, 211]
4‐Octyl itaconate (and itaconate derivatives)	Michael acceptor metabolite derivatives	comparable activity to glyburide in some assays	Covalent modification of NLRP3 (e.g., Cys5) → prevent NEK7 binding	Ischemia/reperfusion, TBI, and MI models	Preclinical/tool molecules	[212, 213]
KN3014	Small molecule PYD‐PYD interface inhibitor	PBMC IL‐1β pIC50 ≈ 7.55 (∼14.6 µM reported)	Blocks PYD‐PYD interactions between NLRP3 and ASC → prevents oligomerization	MWS models/inflammasome activation assays	Preclinical tool compound	[214]
CSC‐6	ASC‐oligomerization inhibitor (small molecule)	THP‐1 IC_50_ ≈ 2.3 µM	Specifically inhibits ASC oligomerization (not direct NACHT ATPase)	Sepsis, gout models	Preclinical tool compound	[215]
MC‐ND‐18 (NLRP3 degrader/PROTAC‐like)	NLRP3 targeted degrader (degrader scaffold)	DC_50_ ≈ 125 nM (cellular)	Induces ubiquitin‐proteasome degradation of NLRP3 (protein level depletion)	UC models (proof‐of‐concept)	Preclinical, proof‐of‐concept degrader	[162]
Compound 8 (PET tracer)	Radiolabeled small molecule (imaging probe)	µM binding/tracer performance	PET imaging ligand for NLRP3 (target engagement)	In vivo imaging of NLRP3 activation/PD readout	Research tool (imaging)	[216]
Compound 13a (fluorescent probe)	Fluorescent imaging probe	RAW264.7 EC_50_ ≈ 49 nM (reported)	Imaging & screening probe for NLRP3	Cellular imaging, probe assays	Research tool	[217]
Glyburide (glibenclamide)	Sulfonylurea (repurposed)	BMDM IC_50_ ≈ 13 µM	Indirect NLRP3 suppression (mechanism partly off‐target)	Diabetes models; early inflammasome discovery work	Marketed (diabetes); repurposing interest	[172]
Tranilast	Anthranilic acid derivative (repurposed)	reported tens µM in vitro	Blocks NLRP3 oligomerization (binds NACHT)	Fibrosis, CAPS models; small clinical studies	Clinical repurposing: Phase II trials ongoing for inflammasome indications	[218, 219]
HT‐6184 (NEK7 allosteric inhibitor)	NEK7 allosteric small molecule (patent scaffold)	company/patent early PK data	Targets NEK7 to block NEK7‐NLRP3 interaction	Chronic inflammation models (preclinical rationale)	Phase I initiated (NCT05447546)	[220]
VENT‐02/VENT series	Brain‐penetrant small molecules (novel scaffold)	nM–µM range in vitro (company data)	NACHT/NLRP3 inhibition with CNS penetration	PD/AD preclinical models; healthy volunteer Phase I completed	Early clinical candidate (Phase I)	[198]
YQ128 (lead)	Small molecule (MCC950‐like analog)	nM–low µM in vitro (company reports)	NACHT binding; brain‐penetrant variants reported	Neuro models (AD)	Preclinical to early clinical planning	[198]
JT002 (or JT‐series)	Small molecule NACHT inhibitor	reported inhibitory readouts (company)	NLRP3 ATPase/inflammasome inhibition	Healthy volunteer studies/early development	Early clinical/preclinical	[198]
RRx‐001 analogue 149‐01	Nitro‐bearing derivative (RRx‐001 analogue)	∼180–200 nM (BMDMs)	Covalent modification (Cys409) → blocks NEK7 binding	Peritonitis, EAE	Preclinical/analog development	[221]
Auranofin/other repurposed kinase/BTK drugs	Approved drugs with NLRP3‐modulating activity	reported in various cell assays	Indirect NLRP3 suppression (NF‐κB/redox/BTK effects)	NAFLD, inflammatory diseases (preclinical/clinical)	Marketed (other indications); repurposing for the inflammasome suggested	[198]
Disulfiram	Repurposed FDA‐approved drug	µM range (THP‐1/BMDM)	Blocks NLRP3 palmitoylation at Cys126, preventing TGN localization and inflammasome assembly	NLRP3‐driven inflammation models	Approved (alcohol use disorder); repurposing candidate for NLRP3‐driven diseases	[188]

Abbreviations: AD, Alzheimer's disease; CAPS, Cryopyrin‐associated periodic syndrome; EAE, experimental autoimmune encephalomyelitis; IBD, inflammatory bowel disease; MAD, multiple ascending dose; MI, myocardial infarction; MSU, monosodium urate; MWS, Muckle–Wells syndrome; NAFLD, non‐alcoholic fatty liver disease; NASH, non‐alcoholic steatohepatitis; OA, osteoarthritis; PD, Parkinson's disease; PD, pharmacodynamics; PK, pharmacokinetics; RCT, randomized controlled trial; SAD, single ascending dose; TBI, traumatic brain injury; UC, ulcerative colitis.

#### 4.4.1 NACHT‐Domain Inhibitors (Sulfonylurea and Non‐Sulfonylurea Scaffolds)

##### 4.4.1.1 Sulfonylurea Derivatives

The sulfonylurea family represents the historical starting point of NLRP3 inhibition. Glyburide (glibenclamide), a sulfonylurea antidiabetic drug, was the first small molecule reported to suppress NLRP3 activity by blocking IL‐1β release (2001, Pfizer) — years before NLRP3 itself was fully identified. Its mechanism is indirect, involving upstream ionic fluxes and metabolic modulation rather than direct inhibition of NLRP3 ATPase activity. Nevertheless, this proof‐of‐concept demonstrated that NLRP3 activity is druggable [172].

The real breakthrough came with MCC950 (2015), a diaryl‐sulfonylurea, that directly binds the NACHT ATPase domain, stabilizing the inactive conformation and preventing oligomerization [60, 183, 222, 223]. Structural studies (e.g., NP3‐146/PDB:7ALV, MCC950 complexes with NLRP3/PDB:7PZC, 7VTQ) revealed how sulfonylurea motifs establish hydrogen‐bond/ionic interactions with residues near the Walker A/B motifs and occupy hydrophobic pockets between WHD/HD1/HD2, thereby conferring exquisite selectivity for NLRP3 over NLRC4 [56, 174, 224]. MCC950 demonstrated sub‐nanomolar potency, excellent selectivity, and robust in vivo efficacy. However, its clinical development in RA was discontinued due to hepatotoxicity, a liability associated with the metabolic instability of the sulfonylurea core [225, 226].

Building on MCC950, medicinal chemists pursued multiple optimization routes. For example, metabolic stabilization by replacing labile furan or benzylic motifs with more robust scaffolds [227, 228]. Electronic modification of the sulfonylurea group (sulfoximine, N‐cyano sulfoximine‐urea, hybrid scaffolds) to reduce oxidative metabolism and improve membrane permeability [227, 229, 230, 231, 232, 233, 234]. Brain penetration design, exemplified by Inzomelid and NT‐0796, for CNS indications [199]. Representative molecules advancing to the clinic include DFV890 (IFM‐2427, Novartis; phase I/II, showing favorable PK and short‐term tolerability), Inzomelid (Roche, CNS‐penetrant), NT‐0796 (NodThera, CNS indications), and Somalix/selnoflast (Inflazome → Roche; multiple indications including CAPS, CAD, PD, COPD, UC) (Table 1) [198, 235, 236, 237, 238].

Despite these advances, metabolic liabilities remain difficult to circumvent. A striking example is GDC‐2394, a Genentech pyrazoloxazine/sulfonamide derivative that replaced earlier furan scaffolds. Structural evidence confirmed high‐affinity binding to NACHT (PDB:8ETR). In first‐in‐human studies, GDC‐2394 demonstrated dose‐proportional PK and potent inhibition of IL‐1β/IL‐18 release [176]. However, in a subsequent drug–drug interaction trial, two participants experienced grade‐4 drug‐induced liver injury (DILI), leading to immediate termination of the program. Both recovered, but the incident highlights a critical translational lesson: potency and structural validation cannot substitute for rigorous early metabolic and hepatotoxicity evaluation [175, 176].

##### 4.4.1.2 Non‐Sulfonylurea Scaffolds

To overcome sulfonylurea liabilities, new NACHT‐targeted scaffolds have been developed, greatly expanding the chemical space:

**NP3 series**. The NP3 series, exemplified by NP3‐562, represents a class of tricyclic/pyridazine‐based small molecules that bind a unique pocket in the NACHT domain, distinct from the canonical MCC950 site. Structural studies, including X‐ray crystallography, confirm this alternative binding mode. NP3‐562 effectively suppresses IL‐1β release in human whole blood assays and demonstrates oral bioavailability in preclinical animal models, with robust efficacy in inflammatory disease settings. Preliminary toxicological studies suggest a favorable safety profile, although comprehensive evaluations are still pending. This series exemplifies how exploring alternative binding sites can expand the chemical space of NLRP3 inhibitor development and provide opportunities for novel drug candidates [203].

**P33**. P33, an optimized derivative of the Ex‐63 scaffold, is a tricyclic/diphenylamine‐based inhibitor that directly engages the NACHT domain of NLRP3. By preventing ASC oligomerization, P33 blocks caspase‐1 activation and subsequent release of IL‐1β and IL‐18. It exhibits high binding affinity (KD ≈ 17.5 nM) alongside favorable oral bioavailability in animal models. Early toxicology studies indicate a good safety margin, supporting its potential for further development. P33 illustrates how systematic chemical optimization of existing scaffolds can yield compounds with improved pharmacokinetic and pharmacodynamic properties, thereby bridging the gap from chemical probe to translatable therapeutic candidate [204].

**SN3‐1**. SN3‐1 is a novel tricyclic scaffold designed using deep learning–assisted computational approaches. Crystallographic analyses reveal that SN3‐1 binds with high affinity (KD ≈ 6.9 nM) to the NACHT domain of NLRP3, occupying an inter‐subdomain pocket. Functionally, it potently blocks inflammasome activation without affecting other inflammasomes such as AIM2 or NLRC4. Preclinical pharmacokinetic studies demonstrate oral bioavailability and favorable absorption and distribution, while toxicological evaluations in both acute and chronic models suggest a promising safety profile. As an exemplar of integrating artificial intelligence with structural biology, SN3‐1 underscores the potential of modern computational tools in scaffold innovation and rational drug design [182, 239].

**OLT1177 (Dapansutrile)**. OLT1177 is a β‐sulfonyl nitrile small molecule structurally distinct from traditional sulfonylureas. It inhibits downstream NLRP3 inflammasome signaling by suppressing ASC oligomerization and caspase‐1 activation, thereby interfering with inflammasome assembly, including ASC speck formation [200]. While some studies suggest that OLT1177 may target the NACHT domain of NLRP3, no crystallographic or structural evidence has definitively confirmed this, and its precise molecular target remains controversial [240]. In preclinical and clinical studies, oral formulations of OLT1177 have shown favorable pharmacokinetics, acceptable safety, and improvements in inflammatory biomarkers in multiple Phase I and II trials (e.g., gout, heart failure, and OA; NCT02134964, NCT02104050, NCT01636141, NCT01768975, and NCT07157735) [200, 201, 202, 241, 170]. To date, no severe hepatotoxicity or major organ toxicity has been reported in these small‐scale trials, although rare or long‐term adverse events remain to be evaluated. As one of the few non‐sulfonylurea NLRP3 inhibitors tested in humans, OLT1177 exemplifies the translational potential of non‐canonical NACHT or inflammasome assembly–targeting scaffolds with tolerable oral dosing.

**CY‐09**. As one of the earliest NACHT inhibitors, CY‐09 targets the Walker‐A ATP‐binding motif and blocks ATP hydrolysis. It demonstrated low‐micromolar activity in vitro and in murine models, but its pharmacokinetics remain suboptimal. While largely used as a research probe, CY‐09 validated ATP‐pocket engagement as an alternative to MCC950's Walker‐B inhibition and serves as proof‐of‐concept for site‐specific NACHT targeting [178, 242].

**Tranilast (Repurposed)**. An anti‐allergic drug already approved for clinical use has been reported to directly bind the NACHT domain of NLRP3 and prevent its oligomerization, thereby suppressing inflammasome activation. With established pharmacokinetics, oral bioavailability, and a well‐documented safety record from decades of clinical use, Tranilast represents an attractive repurposing candidate for inflammatory diseases [218, 219]. However, while its safety profile facilitates rapid clinical translation, further studies are required to validate the selectivity and therapeutic efficacy of NLRP3 targeting in new indications (Table 1).

#### 4.4.2 Covalent and NEK7‐Interface Inhibitors

Covalent modification of the NACHT domain or disruption of the NLRP3–NEK7 interface represents another effective strategy to block inflammasome activation. Compared with reversible ATPase inhibition, covalent strategies typically confer stronger and longer‐lasting inhibition, but they also introduce risks of off‐target modification and irreversible toxicity. In contrast, targeting the NLRP3–NEK7 interface constitutes an “assembly blockade” approach that may theoretically avoid metabolic liabilities associated with direct NACHT inhibition, but raises safety concerns given NEK7's essential role in mitosis.

##### 4.4.2.1 INF series (INF39)

The INF family comprises irreversible Michael acceptor–type inhibitors that covalently modify NLRP3, thereby blocking ATPase activity and disrupting the NLRP3–NEK7 interaction. These compounds demonstrate potent suppression of inflammasome assembly in vitro and efficacy in murine inflammation models. However, their oral bioavailability and pharmacokinetics remain suboptimal, as most remain at the tool compound stage. The electrophilic warhead carries inherent risks of non‐specific protein alkylation, underscoring the need for early chemoproteomics profiling, reactivity optimization, and systematic toxicology studies. To date, no INF‐series compound has advanced beyond preclinical evaluation [208, 209, 243].

##### 4.4.2.2 Oridonin and Derivatives

Oridonin, a natural diterpenoid from *Rabdosia rubescens*, covalently modifies cysteine 279 in the NACHT domain of NLRP3, disrupting NLRP3–NEK7 binding and thereby blocking inflammasome assembly. Oridonin itself suffers from poor oral bioavailability and rapid metabolism; however, derivatives such as compound D6 have been developed with improved potency, selectivity, and efficacy in murine models of acute lung injury. The oridonin lineage exemplifies the “natural product → mechanistic validation → derivative optimization” pathway in drug discovery. While promising, long‐term toxicological and pharmacokinetic validation remains essential [205, 206, 244].

##### 4.4.2.3 Erianin, Costunolide, and Related Natural Covalent Inhibitors

Other natural products, such as erianin (from *Daphne genkwa*) and costunolide (from *Saussurea lappa*), have been reported to covalently modify specific cysteine residues in the NACHT domain (e.g., Cys598), thereby altering ATPase activity and preventing inflammasome assembly. These compounds remain at an early discovery stage, with evidence largely derived from in vitro or murine studies. While natural scaffolds provide unique chemical diversity and privileged reactivity, they typically suffer from poor pharmacokinetics, multi‐target effects, and metabolic instability, requiring rational derivatization for improved drug‐likeness [210, 211].

##### 4.4.2.4 RRx‐001 and Analogues

RRx‐001 is a multifunctional nitroaromatic small molecule that covalently binds cysteine 409 in the NACHT domain of NLRP3, thereby disrupting NLRP3–NEK7 interactions. It also activates Nrf2, providing antioxidative effects. Analogues such as 149‐01 retain potent NLRP3 inhibition with potentially reduced off‐target toxicity. RRx‐001 has been clinically evaluated primarily for oncology indications, where it has demonstrated tolerability across multiple trials, though careful monitoring of off‐target covalent modifications and oxidative stress is warranted (NCT03515538). This lineage illustrates how multi‐functional covalent inhibitors can merge anti‐inflammatory and anticancer mechanisms [221, 245].

##### 4.4.2.5 NEK7 as an Indirect Target (Allosteric/Interface Inhibitors)

Given NEK7's essential role as an adaptor protein in NLRP3 inflammasome assembly, allosteric inhibition of NEK7 offers an indirect strategy to block inflammasome activation. Entrectinib has been shown to interfere with NEK7 residue R121 in vitro, while HT‐6184 (Halia Therapeutics) is a structure‐based allosteric NEK7 inhibitor now in phase I trials. By preventing NEK7–NLRP3 binding, HT‐6184 effectively blocks inflammasome formation without directly targeting NLRP3 itself. This strategy may avoid metabolic liabilities of direct NACHT inhibition, but specificity remains a concern given NEK7's critical role in mitosis, where inhibition could cause cell‐cycle or hematopoietic toxicity [218, 220].

#### 4.4.3 Protein‐Protein Interaction/Assembly Inhibitors

A distinct strategy targets inflammasome assembly rather than ATPase activity. By blocking protein–protein interactions (PPIs)—such as NLRP3–ASC pyrin domain (PYD–PYD) contacts or ASC oligomerization—these molecules disrupt inflammasome formation. This approach theoretically circumvents metabolic liabilities of ATPase inhibitors but is challenged by the flat, featureless nature of PPI interfaces, which complicates drug design.

##### 4.4.3.1 KN3014 (PYD‐PYD Interface Inhibitor)

KN3014 is a piperidine‐containing molecule that selectively blocks the PYD–PYD interaction between NLRP3 and ASC, reducing ASC speck formation and IL‐1β release in human peripheral blood mononuclear cells (PBMCs). Unlike ATPase inhibitors, KN3014 does not interfere with NLRP3's enzymatic function. While effective in cellular assays and IDOL mouse models, its potency is modest (cell‐free IC_50_ ≈ 14.65 µM), and PK optimization is still required. If selectivity can be achieved, PYD‐targeting molecules may offer reduced hepatotoxicity and better tolerability [214].

##### 4.4.3.2 CSC‐6 (ASC Oligomerization Inhibitor)

CSC‐6 binds NLRP3 but primarily inhibits inflammasome activation by preventing ASC oligomerization. In THP‐1 cells, it suppresses IL‐1β secretion (IC_50_ ≈ 2.3 µM), demonstrates good microsomal stability, and shows low cytotoxicity. In murine sepsis and gout models, CSC‐6 alleviates NLRP3‐driven inflammation. However, ASC is shared among multiple inflammasomes, raising potential specificity and safety concerns [215].

##### 4.4.3.3 Endogenous Metabolite

4‐Octyl itaconate (4‐OI), a derivative of the endogenous metabolite itaconate, suppresses NLRP3 inflammasome activation by covalently modifying NLRP3. In mouse NLRP3, 4‐OI modifies Cys548, blocking NLRP3–NEK7 interaction and thereby preventing ASC oligomerization, caspase‐1 activation, and IL‐1β release. In human cells, 4‐OI also inhibits NLRP3–NEK7 binding, though the exact target site is less defined [212, 213]. While effective in vitro and in vivo, 4‐OI has limited pharmacokinetic characterization and is mainly used as a research tool. Its covalent mode of action raises safety concerns, but its origin from an endogenous metabolite may offer some compatibility. This compound highlights metabolite‐derived scaffolds as starting points for inflammasome‐targeted drug discovery.

#### 4.4.4 Key Component Degradation

##### 4.4.4.1 MC‐ND‐18 (Autophagy‐Tethering Degrader)

MC‐ND‐18 is a targeted NLRP3 degrader that operates via an autophagy‐tethering (ATTEC) mechanism: the molecule links an NLRP3‐binding moiety to an LC3/ autophagy‐engaging motif and thereby promotes autophagic clearance of NLRP3 protein in cells. In THP‐1 cells, MC‐ND‐18 shows a cellular DC_50_ of ≈125.5 nM [162]. Compared with classical small‐molecule inhibitors, targeted degraders (PROTACs, ATTECs, molecular glues) offer the advantage of protein removal but often present drug‐likeness challenges (high molecular weight, reduced cell/brain permeability, and limited oral bioavailability). Because NLRP3 contributes to normal host‐defense and tissue homeostasis, sustained or systemic depletion of NLRP3 could increase susceptibility to infection or impair immune responses; therefore, careful dose selection, therapeutic window definition, and indication choice are essential. Overall, MC‐ND‐18 exemplifies the shift from inhibition to protein knockdown as a promising strategy to more durably and specifically modulate the NLRP3 inflammasome.

#### 4.4.5 Upstream/Downstream Interventions and Translational Tools

Finally, several upstream and downstream modulators, as well as imaging tools, provide complementary strategies for modulating NLRP3 biology.

##### 4.4.5.1 Upstream redox/Mitochondrial Protectants

Because mitochondrial dysfunction and ROS represent recurrent upstream convergence points for NLRP3 activation, agents that preserve mitochondrial homeostasis and dampen oxidative stress may serve as alternative or adjunct strategies, potentially mitigating liabilities linked to electrophilic/covalent pharmacology or reactive metabolite formation. Representative phytochemicals with long‐standing dietary/medicinal exposure, including theaflavin, baicalin, and scutellarin, have been reported to attenuate NLRP3‐related inflammatory readouts in cellular and/or in vivo models, often in parallel with improved redox or mitochondrial indices [246, 247] These compounds are not NLRP3‐selective drugs and their pleiotropic targets, achievable exposure in humans, and long‐term safety at therapeutic dosing require further clarification; nevertheless, they highlight a practical upstream node summarized in Table S1.

##### 4.4.5.2 Downstream Inhibitors and Biologics

Compounds such as VX‐765 (caspase‐1 inhibitor) and disulfiram (GSDMD inhibitor) act downstream of NLRP3, while biologics such as anakinra (IL‐1R antagonist), canakinumab (anti–IL‐1β), and rilonacept (IL‐1 trap) have already achieved clinical validation. These agents often serve as bridging or adjunct therapies, providing efficacy in acute or systemic inflammatory settings (Table 1).

##### 4.4.5.3 Imaging Probes and Clinical Tools

Small‐molecule probes such as Compound 8 (a PET tracer with a 1,2,3‐triazole scaffold) and Compound 13a (a fluorescent probe) have been developed to monitor NLRP3 engagement in vitro and in vivo [216, 217]. These pharmacodynamic tools provide crucial data on target occupancy, tissue distribution, and biodistribution, helping bridge preclinical efficacy with clinical pharmacology.

## 5 Conclusion and Perspective

The translational path of NLRP3 inhibitors has revealed that clinical success depends not only on discovering potent chemical matter but on the early integration of pharmacokinetics, toxicology, structural insights, and biomarker validation. Lessons from the clinical discontinuation of MCC950 and GDC‐2394 due to hepatotoxicity highlight the critical need for parallel evaluation of metabolism and toxicity at the preclinical stage. Incorporating human liver microsome profiling, hepatocyte stress assays, bile acid transporter assessments, and early metabolite identification into the discovery‐to‐development continuum should become standard practice, enabling the early elimination of liabilities that otherwise derail clinical translation.

Equally important is the shift toward mechanism‐informed development. Traditional reliance on THP‐1 LPS/ATP models, while useful for throughput, is insufficient to capture the heterogeneity of inflammasome regulation across cell types and species. The use of PBMCs, primary macrophages, and induced pluripotent stem cell models carrying CAPS mutations has demonstrated greater predictive power for human translation. In parallel, structural biology has advanced from fragmentary domain information to near‐complete inflammasome assemblies, allowing rational optimization of NACHT‐binding molecules and early prediction of resistance‐prone variants. Integrating structural data with CAPS mutant binding studies can guide medicinal chemistry away from metabolically soft or reactive liabilities while improving selectivity.

A major determinant of translational success lies in tailoring inhibitors to disease contexts. The highest‐yield indications remain those with clear pathogenic linkage to NLRP3 activation and measurable pharmacodynamic endpoints, such as CAPS, acute gout, certain cardiovascular syndromes, and neuroinflammatory conditions. In these settings, the biological signal is strong, the patient population is molecularly defined, and biomarkers such as ex vivo IL‐1β release, ASC speck imaging, or PET tracers can provide direct readouts of target engagement. In contrast, complex polygenic diseases such as UC pose greater challenges; negative early‐phase results underscore the need for precise patient stratification, disease‐relevant biomarkers, and mechanistic proof‐of‐concept before large trials.

From a chemistry perspective, the field must balance potency with long‐term safety. Covalent or irreversible strategies offer sustained inhibition but raise off‐target and immunogenicity concerns, requiring systematic proteomic profiling and careful optimization. Beyond sulfonylurea derivatives, which provided early scaffolds but also metabolic liabilities, new classes—including interface blockers, atypical scaffolds, and targeted degraders—represent promising alternatives. These approaches expand the pharmacological repertoire and may overcome resistance or safety barriers that hampered earlier candidates.

Looking forward, the most promising trajectory for NLRP3 drug development lies in technology convergence. The integration of AI‐driven compound design, structural biology, high‐content phenotypic screening, and translational biomarker platforms can substantially raise the success rate from probe to clinic. Advances in delivery strategies, including nanocarriers and transdermal systems, together with molecular imaging probes, are expected to be especially transformative for CNS‐directed projects. In this context, the evolution of NLRP3 inhibition is shifting from proof‐of‐concept to precision tailoring—where disease biology, molecular mechanism, and pharmacological design are aligned from the outset. Such a “tailored inhibition” paradigm is likely to define the next generation of safer, more effective inflammasome‐targeted therapeutics.

## Author Contributions

**C.Z**. and **S.J**. contributed equally to the conception and writing of the manuscript. **H.L**. and **K.Z**. assisted in literature collection and discussion. **D.C**. and **G.Y**. supervised the work and revised the manuscript. All authors have read and approved the final manuscript.

## Conflicts of Interest

The authors declare no conflicts of interest.

## Funding

This work was supported by the National Natural Science Foundation of China (No. 22173118, 82304553, 82373064, and 82474009), the Natural Science Foundation of Hunan Province (No.2022JJ80104, 2023JJ40952, and 2024JJ6618), Health Research Project of Hunan Provincial Health Commission (No. 20254677), and Talent Project Established by Chinese Pharmaceutical Association Hospital Pharmacy Department (No. CPA‐Z05‐ZC‐2023‐003).

## Ethics Statement

Not applicable.

## Supporting information




**Table S1**: Summary of representative NLRP3 inflammasome inhibitors, their scaffolds/chemical notes, reported cellular or biochemical potency, proposed mechanism(s) of action, and development status. Evidence codes indicate the type(s) of experimental support available: **(S)** = structural evidence (cryo‐EM/X‐ray; PDB ID provided where available); **(B)** = biochemical evidence (ATPase assay, KD/binding assay); **(P)** = cell‐based, functional, or phenotypic evidence (includes indirect mechanistic readouts and in‐cell assays). For covalent inhibitors, the modified cysteine residue is indicated where reported. Development status is shown where known (Discovery/Preclinical/Phase I/Phase II/Marketing/Repurposed).

## Data Availability

No datasets were generated or analyzed during the current study.
