# Supplementary material for: The NLRP3 Inflammasome: Mechanisms of Activation, Regulation, and Therapeutic Opportunities
Source: MedComm (2020). 2026 Mar 5;7(3):e70660. doi: 10.1002/mco2.70660 (PMC12963795; doi:10.1002/mco2.70660)
Supplement: Supplementary file 1 — Table S1: Summary of representative NLRP3 inflammasome inhibitors, their scaffolds/chemical notes, reported cellular or biochemical potency, proposed mechanism(s) of action, and development status. Evidence codes indicate the type(s) of experimental support available: (S) = structural evidence (cryo‐EM/X‐ray; PDB ID provided where available); (B) = biochemical evidence (ATPase assay, KD/binding assay); (P) = cell‐based, functional, or phenotypic evidence (includes indirect mechanistic readouts and in‐cell assays). For covalent inhibitors, the modified cysteine residue is indicated where reported. Development status is shown where known (Discovery/Preclinical/Phase I/Phase II/Marketing/Repurposed). [file MCO2-7-e70660-s001.docx]

**The NLRP3 Inflammasome:** **Mechanisms of Activation, Regulation, and Therapeutic Opportunities**

Chan Zou^1^*, Shilong Jiang^3,4,5^*, Hui Li^2^, Kai Zhao^6^, Dongshen Cao^2^‡, Guoping Yang^1,2^‡

^1^ Center for Clinical Pharmacology, the Third Xiangya Hospital, Central South University, Changsha, Hunan, 410013, China;

^2^ Xiangya School of Pharmaceutical Science, Central South University, Changsha, Hunan, 410013, China;

^3^ Department of Pharmacy, Xiangya Hospital, Central South University, Changsha, Hunan, 410008, China;

^4^ The Hunan Institute of Pharmacy Practice and Clinical Research, Changsha, Hunan, 410008, China;

^5^ National Clinical Research Center for Geriatric Disease, Xiangya Hospital, Central South University, Changsha, Hunan, 410008, China;

^6^ Department of Hematology and Critical Care Medicine, the Third Xiangya Hospital, Central South University, Changsha, Hunan, China;

*These authors contribute equally.

‡ Address correspondence to: Guoping Yang, Center for Clinical Pharmacology, the Third Xiangya Hospital, Central South University, Changsha, Hunan, 410013, China, Email: [ygp9880@126.com](mailto:ygp9880@126.com); Dongshen Cao, Xiangya School of Pharmaceutical Science, Central South University, Changsha, Hunan, 410013, China, Email: oriental-cds@163.com.

**Table S1** Summary of representative NLRP3 inflammasome inhibitors, their scaffolds/chemical notes, reported cellular or biochemical potency, proposed mechanism(s) of action and development status. Evidence codes indicate the type(s) of experimental support available: **(S)** = structural evidence (cryo-EM / X-ray; PDB ID provided where available); **(B)** = biochemical evidence (ATPase assay, KD / binding assay); **(P)** = cell-based, functional, or phenotypic evidence (includes indirect mechanistic readouts and in-cell assays). For covalent inhibitors the modified cysteine residue is indicated where reported. Development status is shown where known (Discovery / Preclinical / Phase I / Phase II / Marketing / Repurposed).

| **Compound** | **Scaffold/Chemical structure** | Potency(**IC_50_/Activity)** | **Mechanism of Action/Binding sites (Evidence Level)** | Reported Indication(s) | **Development Status** | **Ref** |
| --- | --- | --- | --- | --- | --- | --- |
| MCC950 | 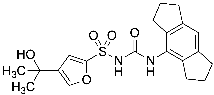 | 8.1 nM (BMDMs), 7.5 nM (PBMCs) | Inhibits NLRP3 NACHT ATPase activity (S/B/P) | >100 preclinical disease models | Phase II (terminated), clinical analogues detailed in table 1 | PDB:7VTQ，7PZC？^1, 2^ |
| NP3-146 |  | Not disclosed | NLRP3 NACHT, inhibiting ATPase activity (S/B/P) | Peritonitis | Preclinical | MCC950 analog，PDB： 7ALV^3^ |
| SN3-1 |  | \| 8 nM (THP-1) \| \| --- \| | NLRP3 NACHT (S/B/P) | Peritonitis, GA, AD | Preclinical | MCC950 analog，KD=6.9 nM，PDB: 8ZEM^4^ |
| DFV890 |  | 12 nM (THP-1) | NLRP3 NACHT (S/B/P) | Gout | Phase II | MCC950 analog，PDB 9HG4^5^ |
| NP3-562 |  | 214 nM (human whole blood) | NLRP3 NACHT (S/B/P) | Peritonitis | Preclinical | (KD= nM) (structural PDB ID: [8RI2](https://www.rcsb.org/structure/8ri2)/KD)^6^ |
| Compound 32 |  | Not disclosed |  |  | Preclinical | 8WSM (cpd32)， |
| NP3-253 |  | 8nM (human whole blood) | NLRP3 NACHT (S/B/P) | Peritonitis，neuroinflammation | Preclinical | 9GU4,and cpd 1(9MGY) 9MIG,9MIE^7^ |
| GDC-2394 |  | \| 63 nM (BMDMs), 51 nM (THP-1) \| \| --- \| | NLRP3 NACHT (S/B/P) | Peritonitis, GA | Phase I | MCC950 analog，PDB ID：8ETR^8^ |
| Diarylsulfonylurea derivative 13a |  | 49 nM (RAW264.7) | NLRP3 NACHT (P) | Fluorescent, imaging probes | Preclinical | MCC950 analog^9^ |
| NT-0796 |  | 0.32 nM (PBMC) | NLRP3 NACHT (P) | Parkinson disease | Preclinical | MCC950 analog^10^ |
| N14 |  | 25 nM | NLRP3 NACHT (P) | Nonalcoholic steatohepatitis, lethal septic shock, colitis | Preclinical | MCC950 analog^11^ |
| NDT-30805 |  | 13 nM (PBMCs) | NLRP3 NACHT (B) |  | Preclinical | MCC950 analog^12^ |
| sulfonylurea-based Compound 15 |  | 23 nM(THP-1) | NLRP3 NACHT (P) | Peritonitis, diabetic kidney disease | Preclinical | MCC950 analog ^13^ |
| P33 | **** | 2.7 nM (THP-1) | NLRP3 NACHT (B/P) | Peritonitis | Preclinical | KD=17.5 nM^14^ |
| BAL-0028 | **** | -57.5 nM (THP-1) | NLRP3 NACHT (B/P) |  | Preclinical | KD=96 nM ^15^ |
| W16 |  | 0.18 μM (J774A.1) | NLRP3 (P) | [UC](https://www.sciencedirect.com/topics/pharmacology-toxicology-and-pharmaceutical-science/ulcerative-colitis) | Preclinical | KD=27.9 nM^16^ |
| AZD4144 |  | 54nM(in vivo) |  | Sepsis | Preclinical | ^17^ |
| B6 |  | 10.69 nM(BMDM) | NACHT(B/P) | systemic inflammation, [peritonitis](https://www.sciencedirect.com/topics/pharmacology-toxicology-and-pharmaceutical-science/peritonitis), and [colitis](https://www.sciencedirect.com/topics/pharmacology-toxicology-and-pharmaceutical-science/colitis) | Preclinical | KD=34 nM^18^ |
| ASD derivative 10 |  | 3.29 nM(THP-1) | NACHT (P) | melanoma | Preclinical | MCC950 analog^19^ |
| Tanshinone derivative (Compound 27) |  | 65 nM (peritoneal macrophages) | NLRP3 NACHT (P) | Sepsis | Preclinical | KD=5.87 μM^20^ |
| YQ128 |  |  |  |  | Preclinical |  |
| Compound 19 |  | 0.12μM (J774A.1) | NLRP3 (P) |  | Preclinical | KD=84 nM^21^ |
| Compound15z | **** | 0.13 μM | NLRP3 NACHT (P) | UC | Preclinical | KD=102.7 nM^22^ |
| Chalcone analogues 14 |  | 251.1 nM (THP-1) | NLRP3 NACHT？ (P) | Peritonitis | Preclinical | ^23^ |
| Z48 |  | 0.26 μM (THP-1) |  | [UC](https://www.sciencedirect.com/topics/pharmacology-toxicology-and-pharmaceutical-science/ulcerative-colitis) | Preclinical | *KD* = 1.05 μM^24^ |
| Isoalantolactone-Based Derivative 49 | **** | 0.29 μM (THP-1) | NLRP3 NACHT covalent modification at D6Cys279 (P) | UC | Preclinical | ^25^ |
| L38 |  | \|  \| \| --- \|  \| 0.32 μM (THP-1) \| \| --- \| | NLRP3 NACHT (P) | UC | Preclinical | ^26^ |
| JC124 | **** | 0.55 μM | NLRP3 NACHT (P) | AD, MI | Preclinical | ^27, 28^ |
| H28 | **** | 0.57 μM (J774A.1) | NLRP3 NACHT(B/P) | Peritonitis | Preclinical | KD=1.15 μM^29^ |
| Tabersonine |  | 0.71 μM (BMDMs) | NLRP3 NACHT (P) | Peritonitis, ALI, sepsis | Preclinical |  |
| Tanshinone 5m |  | 0.84 μM (mouse peritoneal macrophages) | NLRP3 NACHT (P) | Sepsis, NASH | Preclinical | KD=1.34 μM^30^ |
| HNW005 |  | 1.7 μM(mouse peritoneal macrophages) | NLRP3 NACHT (P) | GA | Preclinical | KD=204.6 nM^31^ |
| 7a |  | 1.83 μM (J774A.1) | NLRP3 NACHT? (P) |  | Preclinical | ^32^ |
| Compound 8 | **** | 0.55 μM | PET imaging probe, indirect functional readout (P) | PET imaging probe (indirect / functional readout) | Preclinical | ^33^ |
| S-9 | **** | 3.41 μM (THP-1) | NLRP3 NACHT and NLRP3-PYD (B/P) | Colitis, neuroinflammation | Preclinical | KD=4.34 μM and 6.82 μM^34^ |
| Britannin | **** | 3.63 μM (BMDMs) | NLRP3 NACHT Arg335 and Gly271 (P) | GA, ALI | Preclinical | ^35^ |
| INF200 | **** | 6.61 μM (THP-1) | NLRP3 NACHT (P) |  | Preclinical | ^36^ |
| Glyburide | **** | 13 μM (BMDMs) | NLRP3 NACHT (P) | Diabetes | Repurposed | ^37^ |
| 4-octyl itaconate |  | Similar efficacy to glyburide | NLRP3 NACHT covalent modification at Cys548, preventing NLRP3-NEK7 interaction (P) | TBI, MI, ALI, acute liver injury | Preclinical | ^38^ |
| Fluoxetine |  | – | NLRP3 NACHT (P) | Age-related macular degeneration | Repurposed | ^39^ |
| HS203873 |  | – | NLRP3 NACHT (P) |  | Preclinical | ^40^ |
| Tetrahydroquinoline compound 6 |  | 7.8 μM(THP-1) | NLRP3 NACHT (P) | Colitis | Preclinical | ^41^ |
| Dehydrocostus Lactone |  |  | NLRP3 NACHT covalent modification at Cys280 (P) | peritonitis | Preclinical | KD= 518 nM^42^ |
| Gymnoasins AC 1 | **** | – | NLRP3 NACHT (B/P) |  | Preclinical | KD=29.34 μM^43^ |
| **N102** | **** | 0.029 μM (THP-1 pyroptosis) | Likely targeting NLRP3 NACHT（P） |  | Preclinical | ^44^ |
| J114 | **** | 0.098 μM (THP-1) | disturbed the interaction of NLRP3 or [AIM2](https://www.sciencedirect.com/topics/pharmacology-toxicology-and-pharmaceutical-science/melanoma) with the [adaptor protein](https://www.sciencedirect.com/topics/pharmacology-toxicology-and-pharmaceutical-science/adaptor-protein) ASC and inhibited ASC oligomerization（P） |  | Preclinical | ^45^ |
| CY-09 | **** | 2.4 μM (BMDMs) | Binding ATP-hydrolysis motif (Walker A motif) (B/P) | IBD, CAPS, T2D | Preclinical | ^46, 47^ |
| Sorbremnoids A |  | 2.11 μM (J774A.1) | NLRP3 Walker A inhibition (P) | Diabetic refractory wound healing | Preclinical | ^48^ |
| Dapansutrile / OLT1177 | 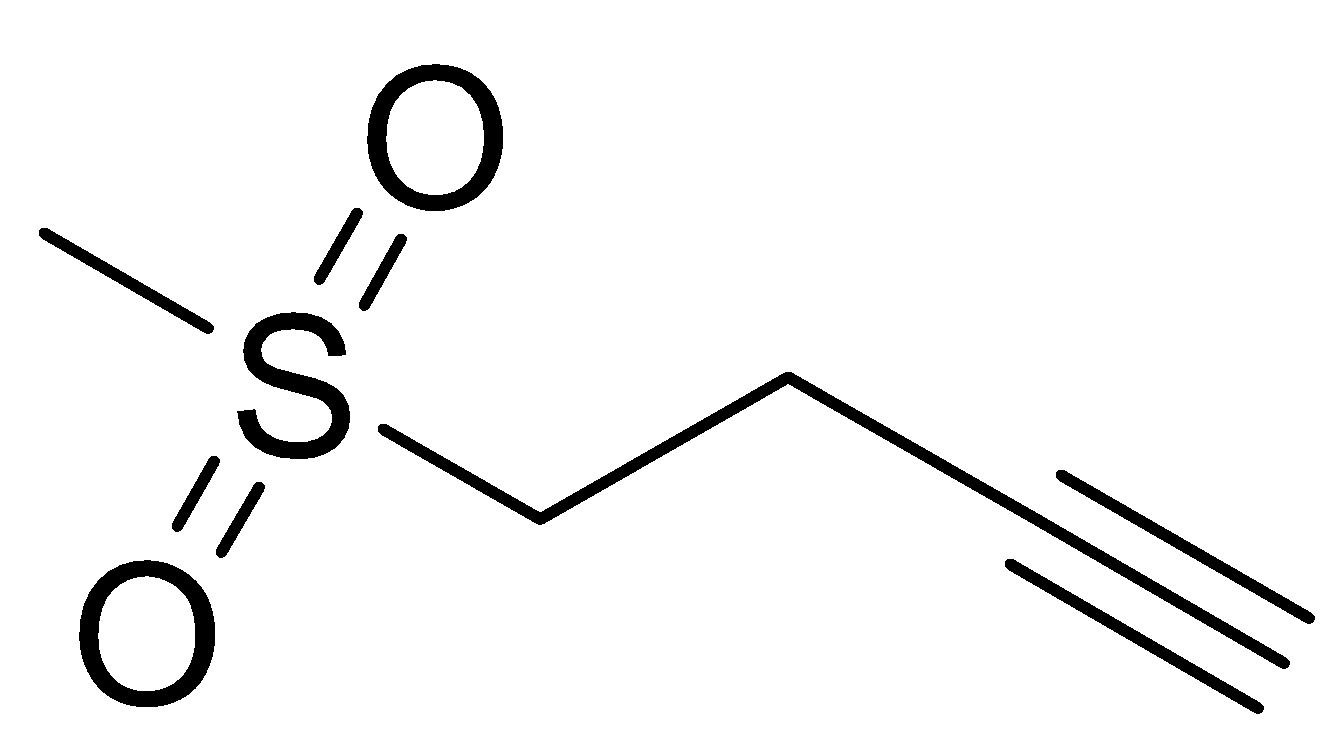 | 1~3μM (J774A.1) | Inhibiting NLRP3 ATPase activity (B/P) | Knee osteoarthritis, gout, AD | phase II | ^49^ |
| Compound 9 |  | 6.9 μM (THP-1) | Inhibiting Caspase-1 and NLRP3 ATPase activity (B/P) |  | Preclinical | ^50^ |
| Pterostilbene-based derivatives 47 | 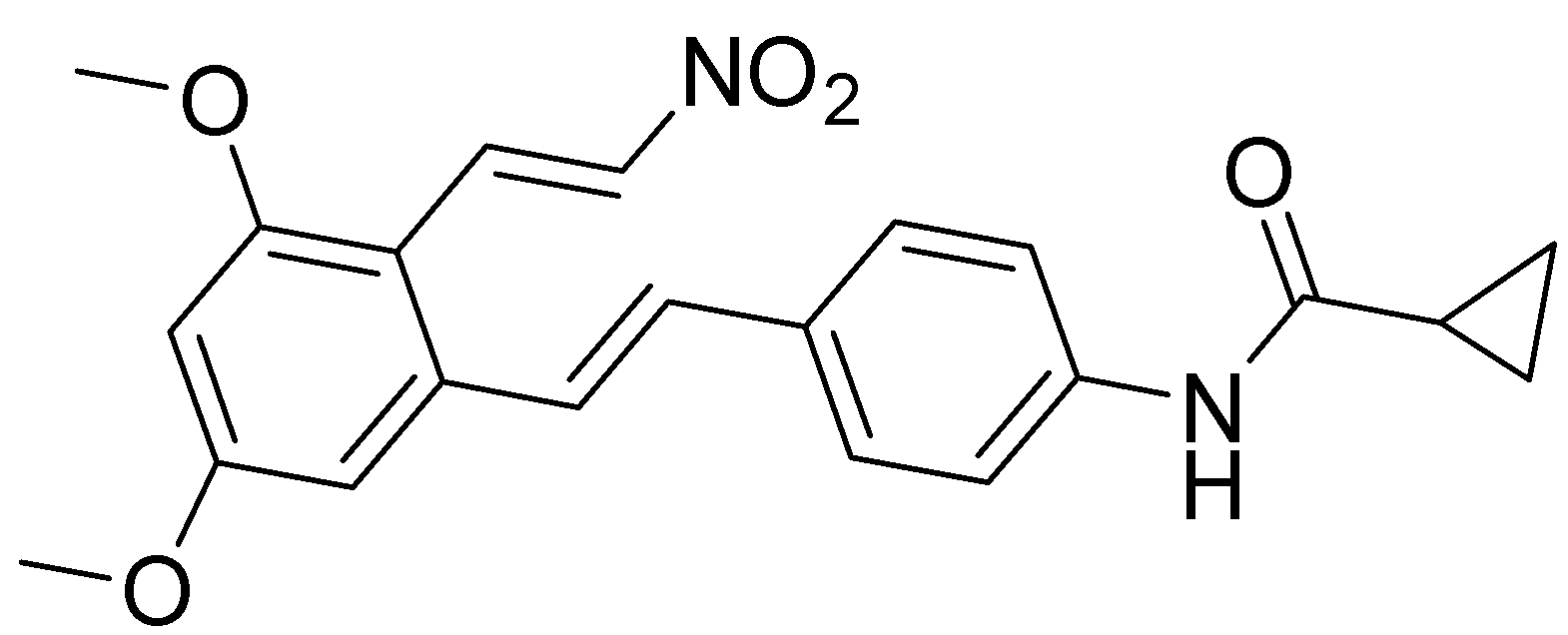 | 0.56 μM | Inhibit NLRP3 ATPase activity (B/P) | Colitis | Preclinical | KD=263 nM^51^ |
| MNS | 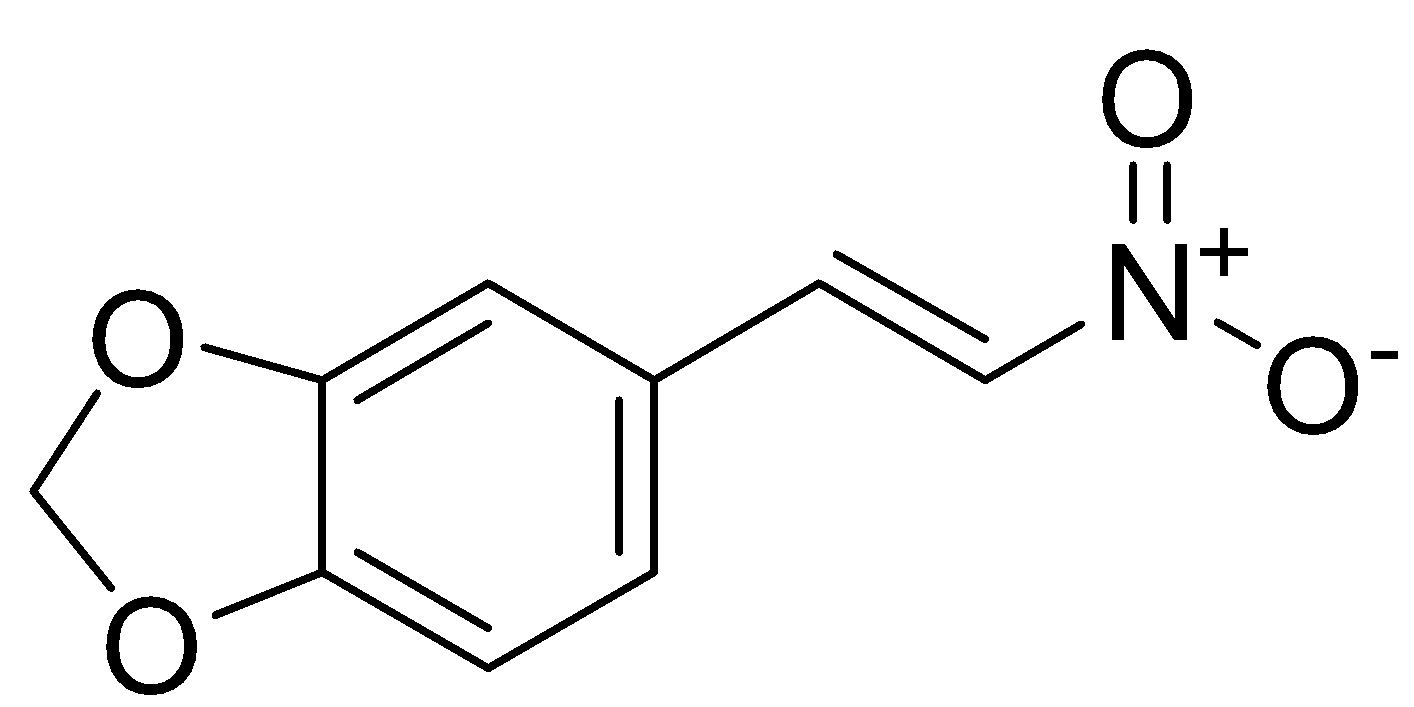 | 2 μM (BMDMs) | Inhibiting NLRP3 ATPase activity (B/P) | Wound healing and osteosarcoma tumor | Preclinical | ^52^ |
| INF39 | 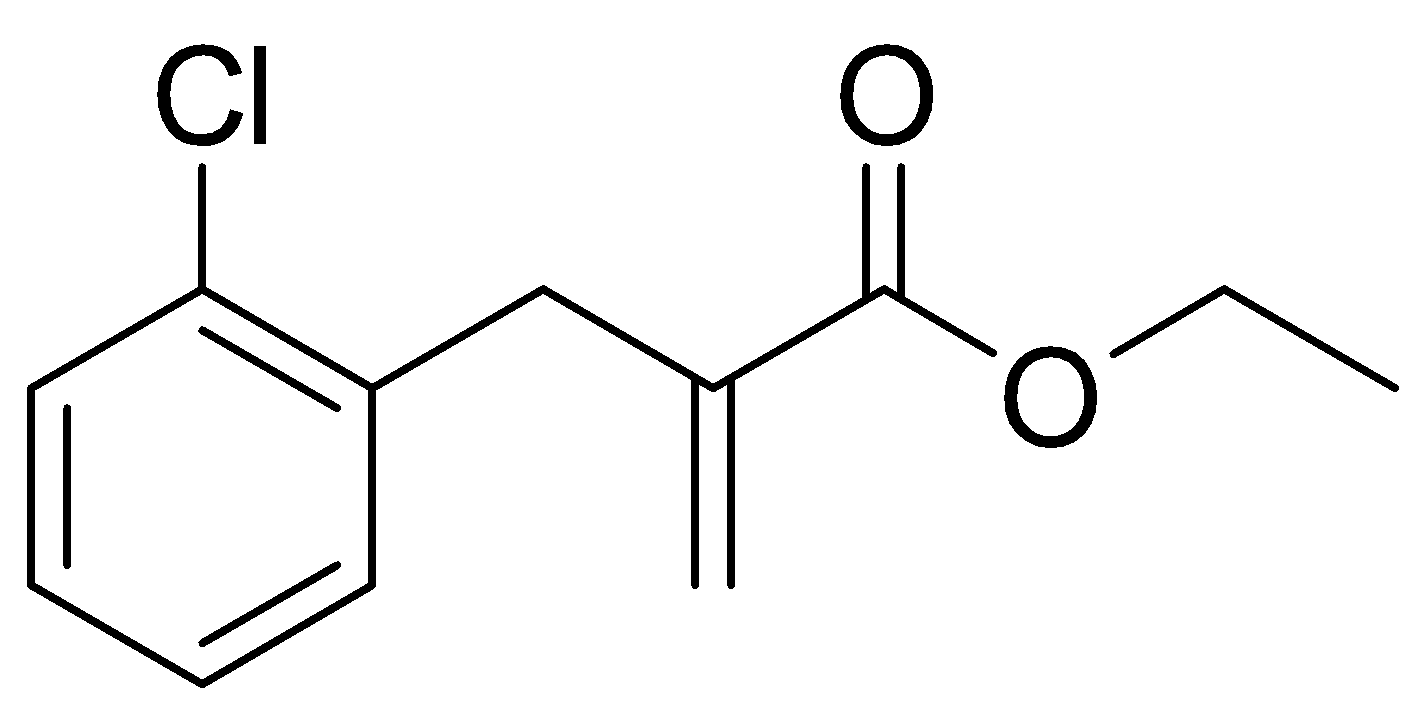 | 10 μM (THP-1) | Inhibiting NLRP3 ATPase and NEK7-NLRP3 interaction (B/P) | IBD | Preclinical | ^53^ |
| Compound 18 (INF172) |  | – | Inhibiting NLRP3 ATPase activity (B/P) |  | Preclinical | ^54^ |
| Erianin | 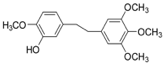 | – | NLRP3 NACHT covalent modification at Cys463, inhibiting ATPase activity (B/P)) | Peritonitis, GA, T2D | Preclinical | KD=50 nM^55^ |
| Costunolide | **** | – | NLRP3 NACHT covalent modification at Cys598, inhibiting ATPase activity (B/P) |  | Preclinical | ^56^ |
| Tivantinib |  | – | c-Met inhibitor / blocking NLRP3 ATPase activity(B/P) | Peritonitis, ALI, EAE | Repurposed(Phase III) | KD=372.64 nM^57^ |
| SB-222200 | **** | – | NLRP3 NACHT, preventing NLRP3-NEK7 interaction (P) | Peritonitis，acute intestinal inflammation | Preclinical | KD=351.8 nM^58^ |
| Oridonin | 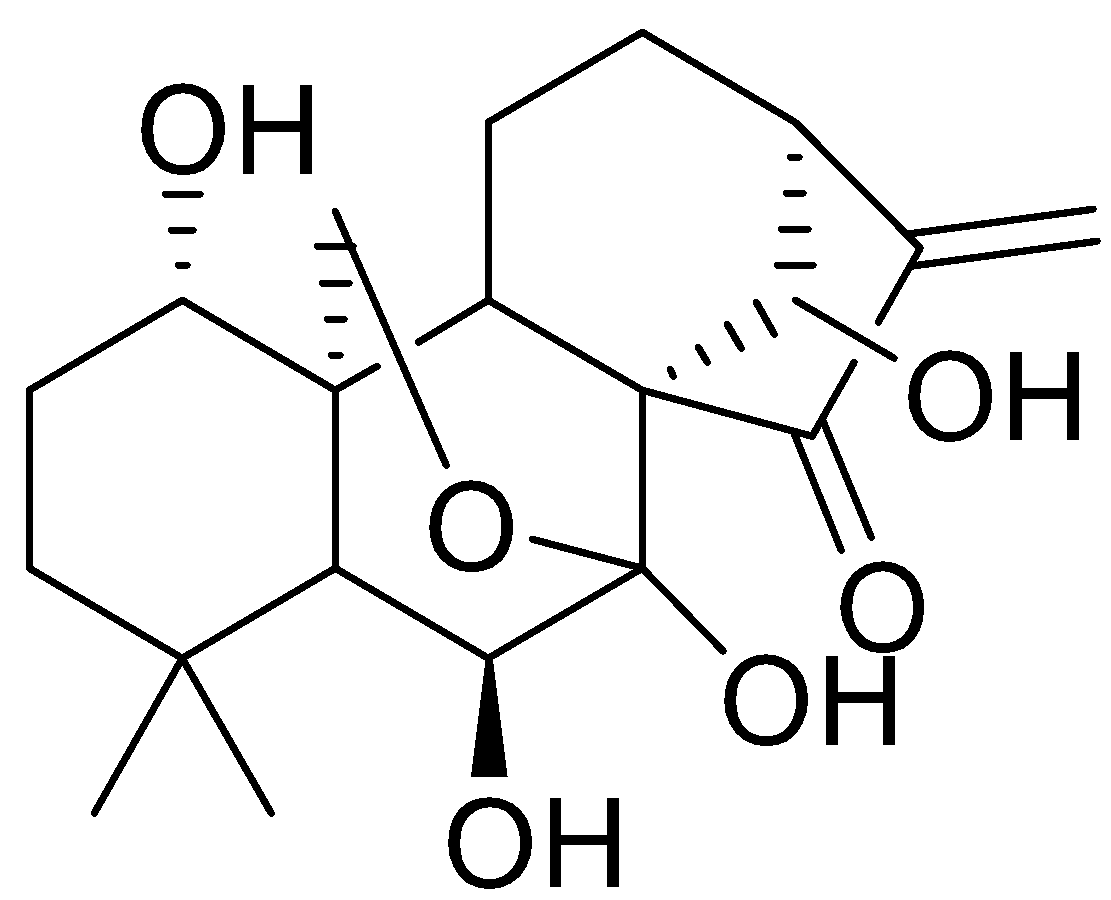 | ~6 μM (BMDMs) | NLRP3 NACHT covalent modification at Cys279, preventing NLRP3-NEK7 interaction (P) | TBI, MI, ALI, acute liver injury | Preclinical | KD=52.5 nM^59, 60^ |
| Oridonin derivatives 32 |  | 77.2 nM (BMDMs) | InhibitingNLRP3-ASC/NEK7 interaction and ASC oligomerization and (P) | GA | Preclinical | ^61^ |
| **D6** |  | 41.79 nM (BMDM) | NLRP3 NACHT (P) | ALI | Preclinical | KD=5.17 μM^62^ |
| RRx-001 | 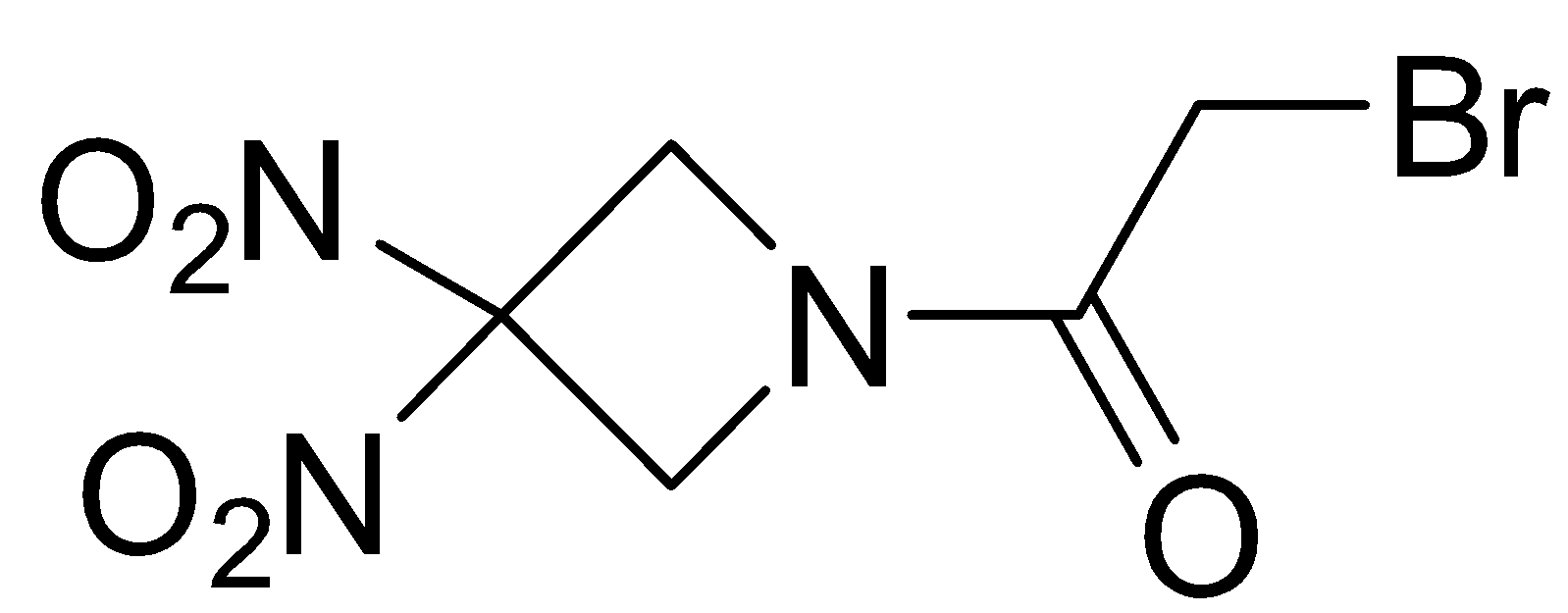 | 200–300 nM (BMDMs) | NLRP3 NACHT covalent modification at Cys409, preventing NLRP3-NEK7 interaction (P) | Allergic asthma, tumors | phase II | KD=20.8 nM^63^ |
| Alantolactone | **** | – | NLRP3 NACHT, preventing NLRP3-NEK7 interaction (P) | ALI, GA | Preclinical | KD=145 μM^64^ |
| Chloranthalactone B | **** | – | NLRP3 NACHT covalent modification at Cys279, preventing NLRP3-NEK7 interaction (P) | Gout, peritonitis, ALI | Preclinical | ^65^ |
| HT-6184 | Undisclosed  (representative patent structure) |  | NEK7, preventing NLRP3-NEK7 interaction (P) | Heart disease, AD, PD fatty liver, cancer, IBD | Phase I |  |
| Berberine (BBR) | 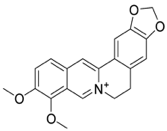 | 5.1 μM (THP-1) | NEK7, preventing NLRP3-NEK7 interaction (P) | T2D, atherosclerosis, NAFLD, neurodegenerative diseases | Preclinical | KD=15.6 μM^66^ |
| Licochalcone B | 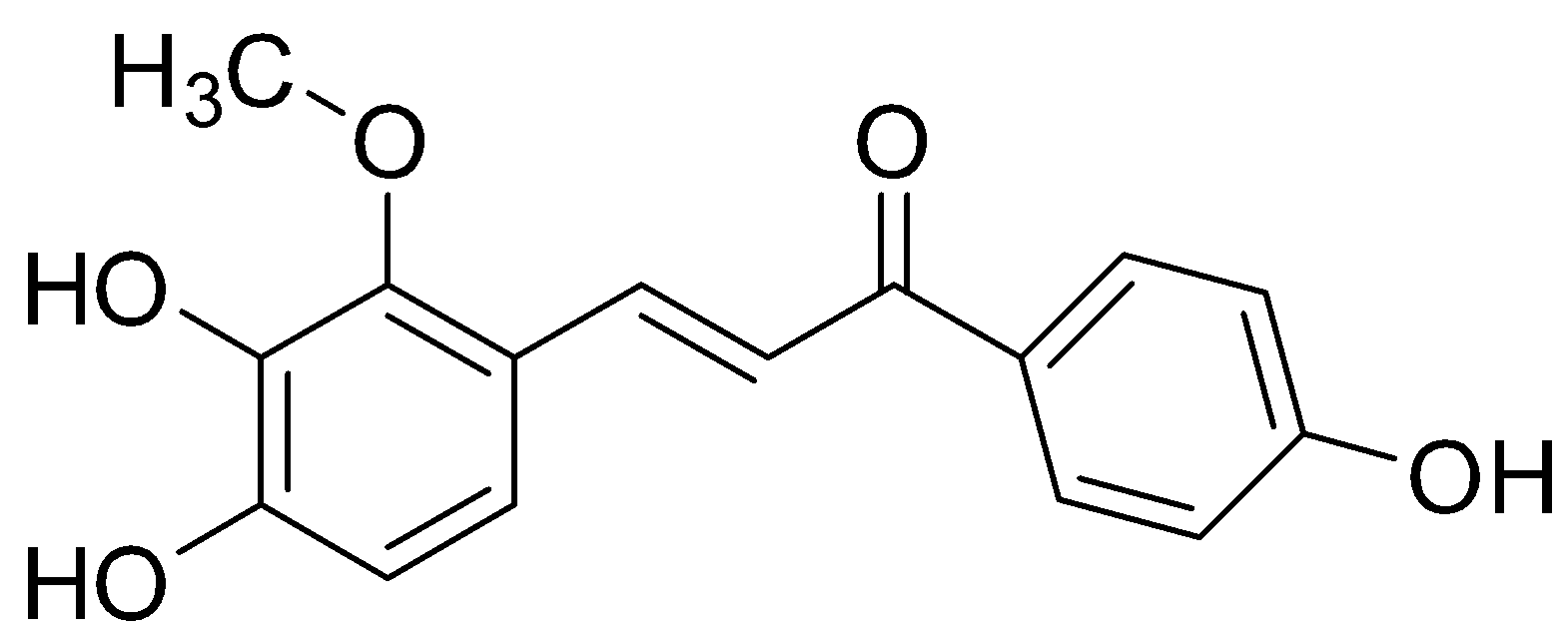 | 18.1 μM (BMDMs) | NEK7, preventing NLRP3-NEK7 interaction and NLRP3-dependent ASC oligomerization (P) | Septic shock, peritonitis, NASH | Preclinical | ^67^ |
| Entrectinib |  | 0.65 μM (BMDM) | NEK7 R121, preventing NLRP3-NEK7 interaction (P) | Peritonitis, diabetes | Preclinical | ^68^ |
| NIC7w |  | 5.5 μM (THP-1) | Inhibiting NEK7-NLRP3 interaction (P) | AD, psoriasis | Preclinical | ^69^ |
| Biaryl urea derivatives Compound **23** |  | 0.048 μM (BMDM) | NEK7, preventing NLRP3-NEK7 interaction (P) |  | Preclinical | ^70^ |
| Rociletinib |  | 0.47 μM (BMDM) | NEK7 covalent modification at Cys79, preventing NLRP3-NEK7 interaction(P) | T2D | Repurposed | KD=6.7 μM ^71^ |
| NK7-902 |  | 9.1nM(hPBMC) | NEK7 degrader (P) |  | Preclinical | ^72^ |
| II-8 | **** | 3.33 μM (THP-1) | NLRP3 LRR (P) | Rheumatoid arthritis | Preclinical | ^73^ |
| Octyl gallate | **** | 0.617 μM (BMDMs) | NLRP3 LRR (P) | Gout, sepsis | Preclinical | ^74^ |
| KN3014 | 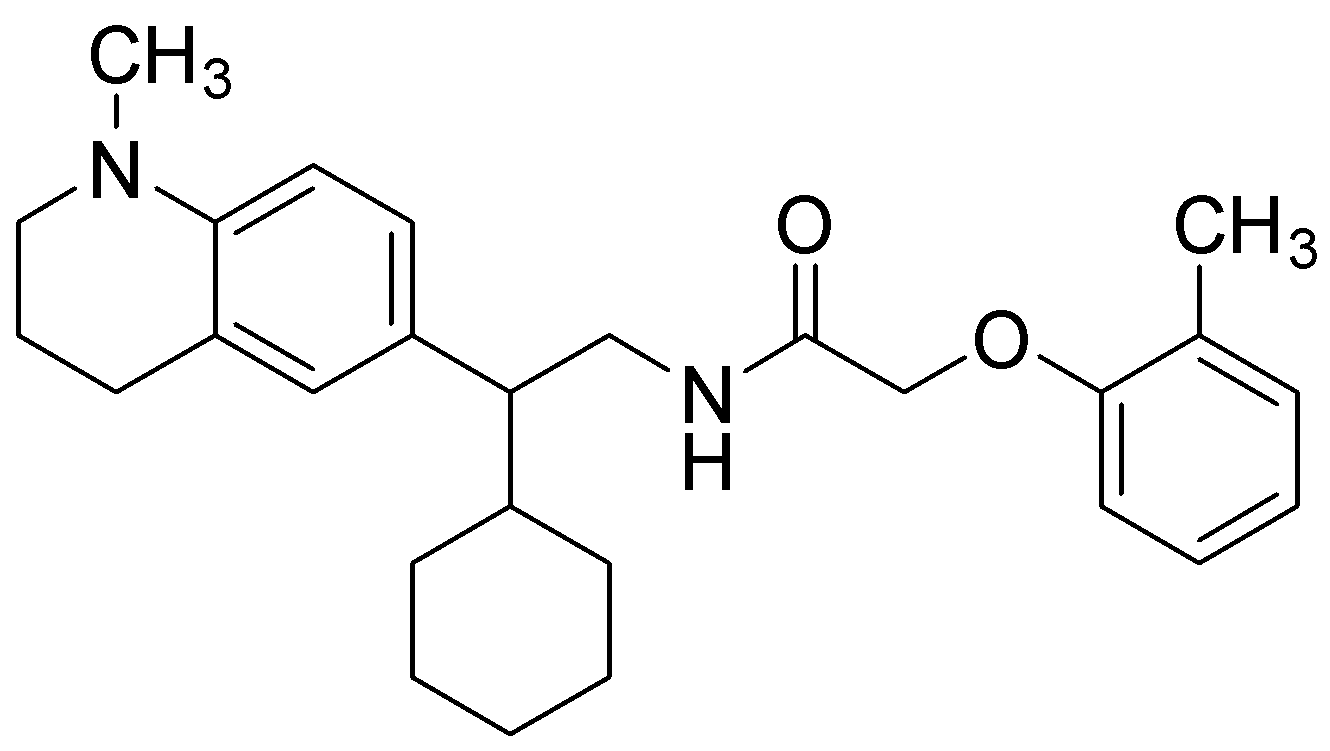 | 14.65 µM (PBMCs) | Inhibit the PYD-PYD interactions between NLRP3 and ASC (P) | MWS | Preclinical | ^75^ |
| QM380 |  | 21.1 μM (THP-1) | NLRP3-PYD, blocking NLRP3 homo-oligomerization (P) |  | Preclinical | ^76^ |
| CSC-6 | **** | 2.3 μM (THP-1) | Specifically binds NLRP3, blocking ASC oligomerization (P) | Sepsis, Gout | Preclinical | ^77^ |
| imidazolidinone derivativescompound 23 | **** | 4.8 μM (THP-1) | NLRP3 (P) | [peritonitis](https://www.sciencedirect.com/topics/pharmacology-toxicology-and-pharmaceutical-science/peritonitis), arthritis | Preclinical | KD=0.815 μM^78^ |
| Tranilast | 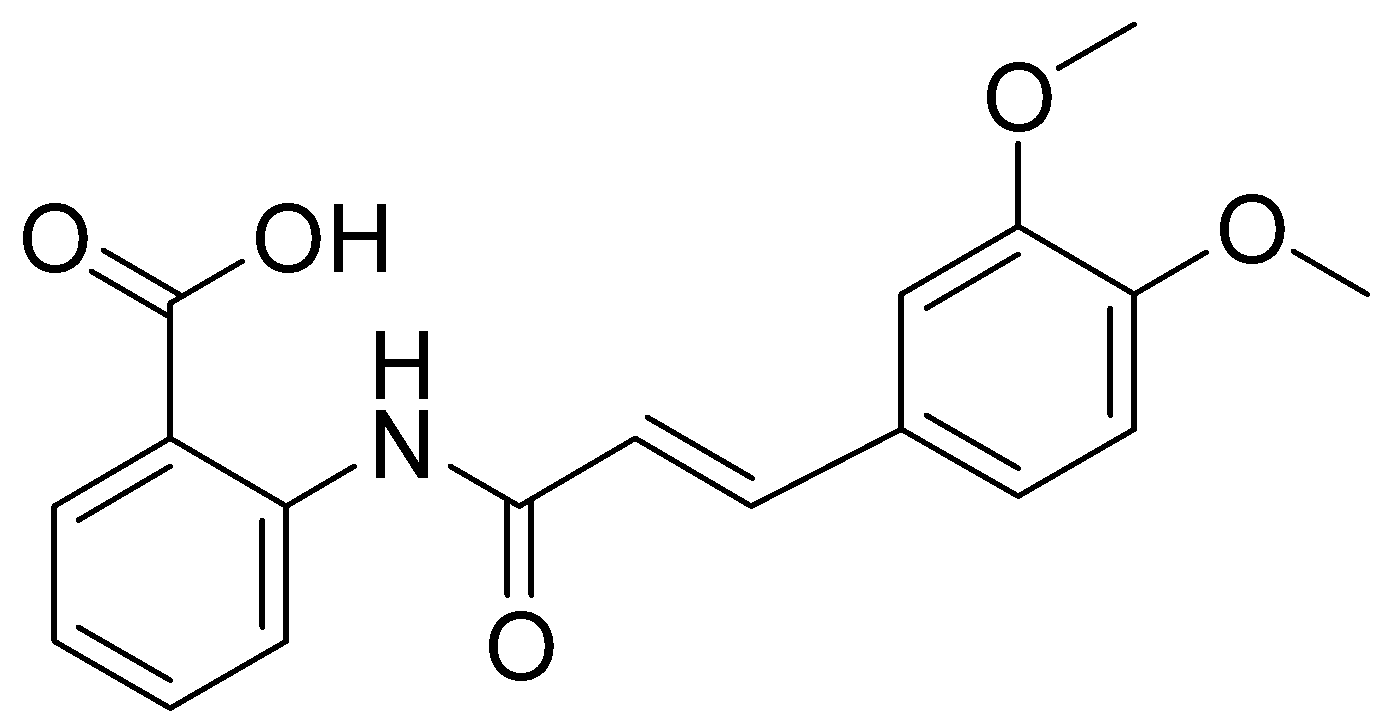 | 25–50 μM | \| Blocking NLRP3 oligomerization by binding NACHT (C, P) \| \| --- \| | CAPS, sarcoidosis, scleredema diabeticorum, mucinoses | Repurposed(Phase II) | ^79^ |
| 8-hydroxfuinoline derivative, compound 10 |  | 1.4 μM(J774A.1) | disrupts key protein-protein interactions, including NEK7-NLRP3, NLRP3−NLRP3, NLRP3−ASC(P) | UC | Preclinical | ^80^ |
| BOT-4-one | 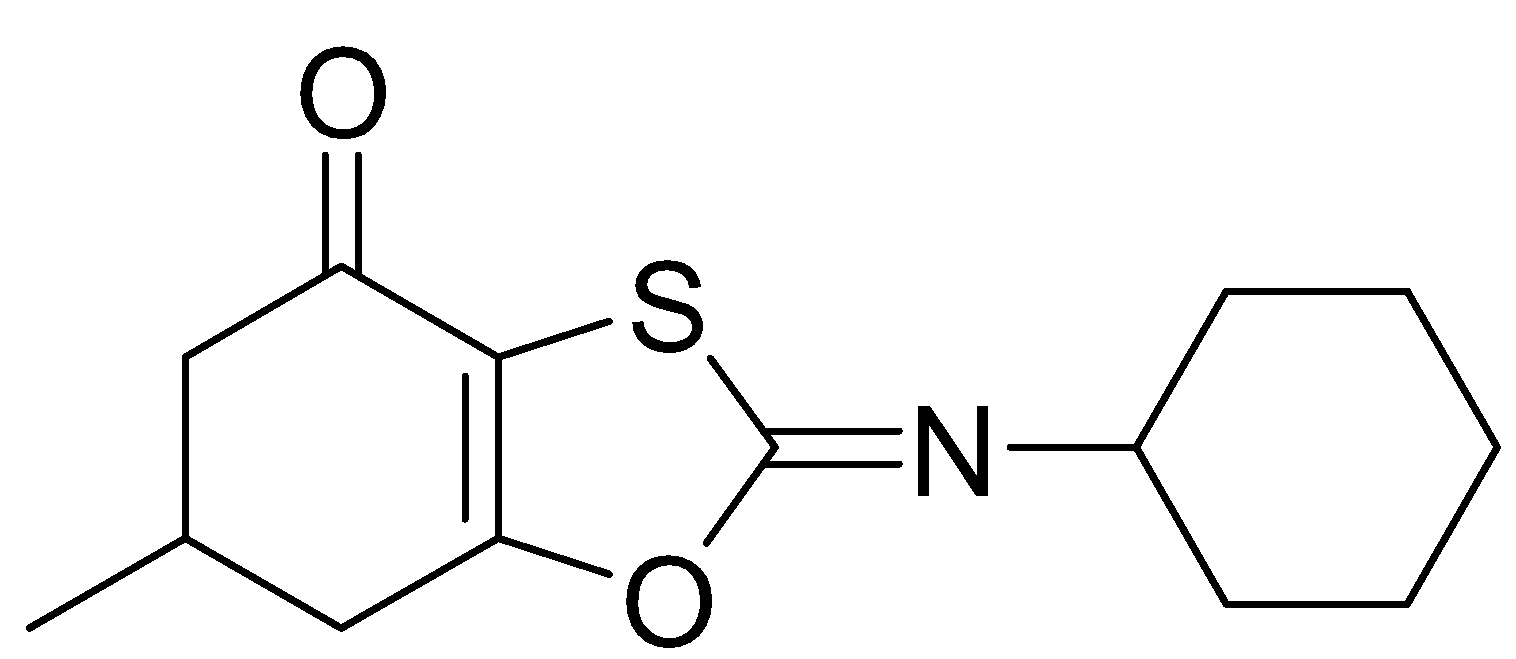 | 0.67 μM | Inhibiting NF-κB dependent NLRP3 expression, increased NLRP3 ubiquitination, inhibiting NLRP3 ATPase activity (P) | Peritonitis | Preclinical | ^81^ |
| Parthenolide | 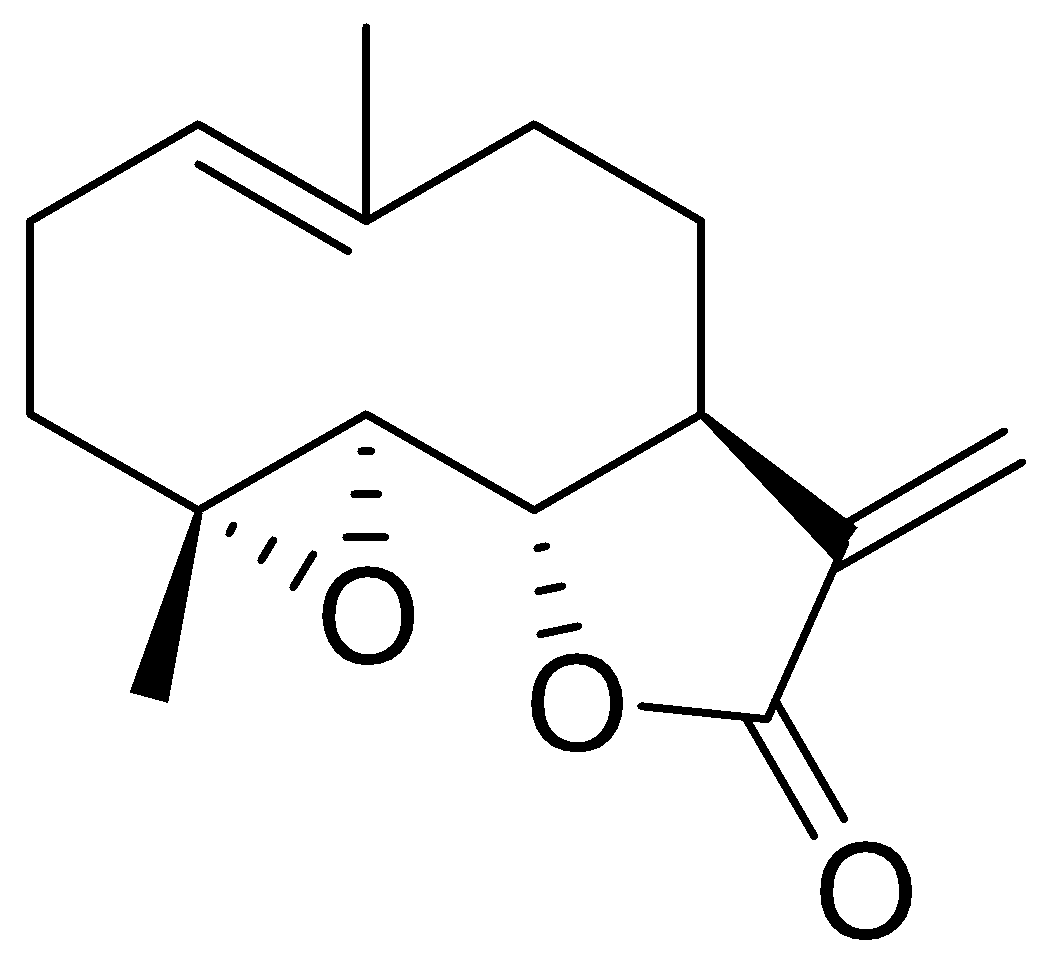 | 1.4 μM (BMDMs) | Inhibiting NF-κB dependent NLRP3 expression, increased NLRP3 ubiquitination, inhibiting NLRP3 ATPase activity (P) | Brain edema, TBI | Preclinical | ^82^ |
| BAY 11-7082 |  | 5 μM (BMDMs) | Inhibiting NF-κB dependent NLRP3 expression, increased NLRP3 ubiquitination, inhibiting NLRP3 ATPase activity (P) | Gout, silicosis, neurodegeneration, periodic fever syndromes | Preclinical | ^82^ |
| Auranofin | 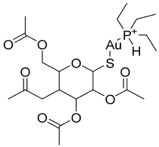 | – | NF-κB/NLRP3 axis and cellular redox system Xc (P) | NAFLD, acne vulgaris | Repurposed | ^83^ |
| Scutellarin | 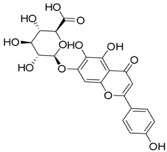 | – | NF-κB/NLRP3 pathway (P) | Idiopathic pulmonary fibrosis | Preclinical | ^84^ |
| NIC-0102 |  | Nanomolar activity in J774A.1 (IL-1β pIC50=7.55) | Promoting NLRP3 polyubiquitination (P) | UC | Preclinical | ^85^ |
| Myricetin | 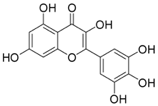 | – | Promoting NLRP3 ubiquitination (P) | NLRP3-driven inflammatory diseases | Preclinical | ^86^ |
| MF-094 | 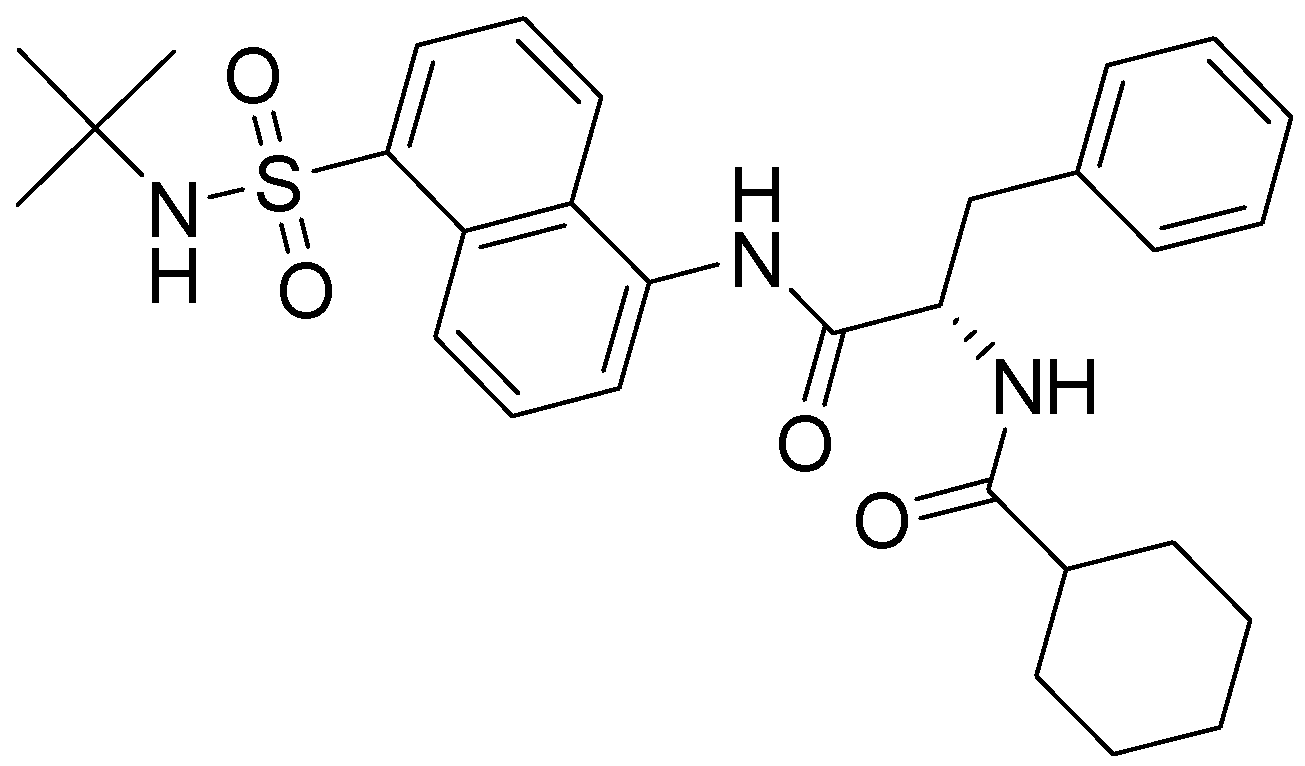 | – | Inhibiting NLRP3 deubiquitination (USP30 inhibitor) (P) | Diabetes | Preclinical | ^87^ |
| Thiolutin |  | 9.69 nM (HMDMs) | Inhibiting NLRP3 deubiquitination (BRISC/BRCC3 inhibitor) (P) | Sepsis, peritonitis, EAE, CAPS, NAFLD | Preclinical | ^88^ |
| MC-ND-18 | **** | DC50=125.5 nM | NLRP3 degrader(P) |  | Preclinical | KD=0.929 μM^89^ |
| Dimethyl fumarate | 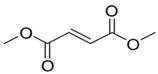 | - | Increasing PKA-dependent NLRP3 phosphorylation (P) | Auto-immune hepatitis | Repurposed | ^90^ |
| Wedelolactone |  | - | Increasing PKA-dependent NLRP3 phosphorylation (P) | Peritonitis, GA | - | ^91^ |
| Ibrutinib |  | - | BTK inhibitor, affecting NLRP3 phosphorylation (P) | Ischaemic brain injury, metabolic inflammation, stress-induced anxiety disorders and endotoxemic lung injury | Repurposed | ^92^ |
| Ruxolitinib |  | - | JAK inhibitor, reduceing NLRP3 activation responses via JAK2/STAT3 pathway (P) | Ischemic stroke | Repurposed | ^93^ |
| Candesartan |  | - | MAPK/NF-κB signaling inhibition (P) | Inflammatory diseases | Repurposed | ^94^ |
| Fc11a-2 |  | Moderate activity | MAPK/STAT1 signaling inhibition(P) | Colitis |  | ^95^ |
| Olaparib |  | - | PARP-1 inhibitor, inhibiting NLRP3 SUMOylation (P) | Asthma,Huntington’s disease, chronic ocular hypertension | Repurposed | ^96^ |
| CIB-1476 |  | 0.65μM (J774A.1) | pro-caspase-1 covalent modification at Cys285 and 397(B/P) | GA | Preclinical | ^97^ |
| β-hydroxybutyrate |  | - | Blocking K⁺ efflux (P) | - | Preclinical | ^98^ |
| 2-APB | 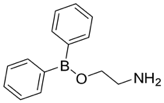 | 67 μM | Affecting Ca²⁺ mobilization (P) | Peritonitis | Preclinical |  |
| NBC6 | 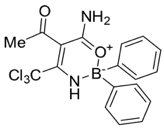 | 574 nM (THP-1) | Affecting Ca²⁺ mobilization (P) | - | Preclinical |  |
| Methyl gallate | 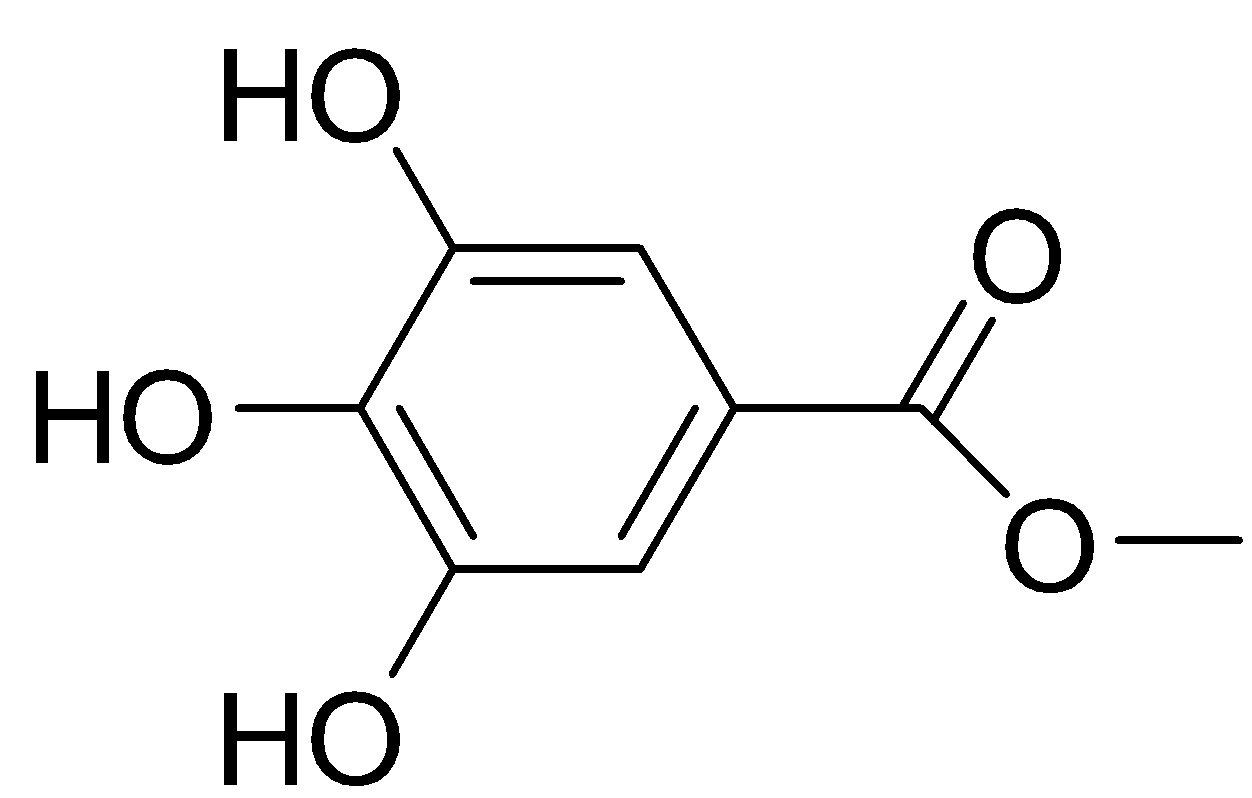 | ~10 μM (BMDMs) | Blocking ROS overgeneration (P) | Hyperuricemia and renal insufficiency, Colitis, arthritis | Preclinical | ^99^ |
| 4-hydroxy auxarconjugatin B |  | 1 μM | Autophagic inducer (P) |  | Preclinical | ^100^ |
| Andrographolide |  | - | Autophagy induction(P) | Colitis, tumor | Preclinical | ^101^ |
| 35m and hydrazide-based derivates |  | 2.61 μM (J774A.1) | HDAC6 inhibition (P) | Endotoxic shock, peritoneal, IBD, psoriasis | Preclinical | ^102, 103^ |
| Baicalin |  | - | Blocking ROS/mitochondrial stress (P) | Sepsis/peritonitis | Preclinical | ^104^ |
| Theaflavin |  | - | Blocking ROS/mitochondrial stress (P) | Acute gouty peritonitis Acute gouty peritonitis | Preclinical | ^105^ |
| Disulfiram |  | - | Blocks NLRP3 palmitoylation,also blocks GSDMD pore formation (P) | Endotoxemia / sepsis models | Repurposed | ^106^ |

**Abbreviations:** UC, ulcerative colitis;AD, Alzheimer’s disease; GA, gouty arthritis; NAFLD, nonalcoholic fatty liver disease; ACS, acute coronary syndromes; NASH, non-alcoholic steatohepatitis; TBI, traumatic brain injury; MI, myocardial infarction; ALI, acute liver injury;T2D,type 2 diabetes; EAE, experimental autoimmune encephalomyelitis; IBD, inflammatory bowel disease; PPAR, peroxisome proliferator-activated receptor; ROS, reactive oxygen species; PKA, protein kinase A; BTK, Bruton's tyrosine kinase.

**Note.** Because many reported NLRP3 modulators act via diverse phenotypic/indirect mechanisms (e.g., redox modulation, ubiquitination, kinase signaling, autophagy), this table is restricted to compounds with relatively well-characterized or representative mechanisms (direct NACHT/ATPase engagement, NEK7–NLRP3 interference, ASC oligomerization blockade, covalent cysteine modification, or well-defined biochemical binding evidence).

Reference:

1. Coll, R.C. *et al.* A small-molecule inhibitor of the NLRP3 inflammasome for the treatment of inflammatory diseases. *Nat Med* **21**, 248-255 (2015).

2. Hochheiser, I.V. *et al.* Structure of the NLRP3 decamer bound to the cytokine release inhibitor CRID3. *Nature* **604**, 184-189 (2022).

3. Dekker, C. *et al.* Crystal Structure of NLRP3 NACHT Domain With an Inhibitor Defines Mechanism of Inflammasome Inhibition. *J Mol Biol* **433**, 167309 (2021).

4. Shi, C. *et al.* Deep-Learning-Driven Discovery of SN3-1, a Potent NLRP3 Inhibitor with Therapeutic Potential for Inflammatory Diseases. *J Med Chem* **67**, 17833-17854 (2024).

5. Shen, D.M. *et al.* Discovery of DFV890, a Potent Sulfonimidamide-Containing NLRP3 Inflammasome Inhibitor (vol 68, pg 5529, 2025). *Journal of Medicinal Chemistry* **68**, 7841-7841 (2025).

6. Velcicky, J. *et al.* Discovery of Potent, Orally Bioavailable, Tricyclic NLRP3 Inhibitors. *J Med Chem* **67**, 1544-1562 (2024).

7. Mackay, A. *et al.* Discovery of NP3-253, a Potent Brain Penetrant Inhibitor of the NLRP3 Inflammasome. *Journal of Medicinal Chemistry* **67**, 20780-20798 (2024).

8. McBride, C. *et al.* Overcoming Preclinical Safety Obstacles to Discover (S)-N-((1,2,3,5,6,7-Hexahydro-s-indacen-4-yl)carbamoyl)-6-(methylamino)-6,7-dihydro-5H-pyrazolo[5,1-b][1,3]oxazine-3-sulfonamide (GDC-2394): A Potent and Selective NLRP3 Inhibitor. *J Med Chem* **65**, 14721-14739 (2022).

9. Zhao, J. *et al.* Synthesis and pharmacological validation of fluorescent diarylsulfonylurea analogues as NLRP3 inhibitors and imaging probes. *Eur J Med Chem* **237**, 114338 (2022).

10. Harrison, D. *et al.* Discovery of Clinical Candidate NT-0796, a Brain-Penetrant and Highly Potent NLRP3 Inflammasome Inhibitor for Neuroinflammatory Disorders. *J Med Chem* **66**, 14897-14911 (2023).

11. Li, Z. *et al.* Novel Sulfonylurea-Based NLRP3 Inflammasome Inhibitor for Efficient Treatment of Nonalcoholic Steatohepatitis, Endotoxic Shock, and Colitis. *J Med Chem* **66**, 12966-12989 (2023).

12. Harrison, D. *et al.* Discovery and Optimization of Triazolopyrimidinone Derivatives as Selective NLRP3 Inflammasome Inhibitors. *ACS Med Chem Lett* **13**, 1321-1328 (2022).

13. Liu, Y.T. *et al.* Discovery of Novel Sulfonylurea NLRP3 Inflammasome Inhibitor for the Treatment of Multiple Inflammatory Diseases. *Journal of Medicinal Chemistry* **68**, 7243-7262 (2025).

14. Fu, Z. *et al.* Discovery of Potent, Specific, and Orally Available NLRP3 Inflammasome Inhibitors Based on Pyridazine Scaffolds for the Treatment of Septic Shock and Peritonitis. *J Med Chem* **67**, 15711-15737 (2024).

15. Wilhelmsen, K. *et al.* Discovery of potent and selective inhibitors of human NLRP3 with a novel mechanism of action. *Journal of Experimental Medicine* **222** (2025).

16. Wu, R.W. *et al.* Discovery, synthesis, and biological mechanism evaluation of novel quinoline derivatives as potent NLRP3 inhibitors. *European Journal of Medicinal Chemistry* **289** (2025).

17. Johansson, A. *et al.* Discovery of AZD4144, a Selective and Potent NLRP3 Inhibitor for the Treatment of Inflammatory Diseases. *Journal of Medicinal Chemistry* **68**, 14195-14222 (2025).

18. Lv, Q. *et al.* Discovery of novel non-sulfonylurea NLRP3 inflammasome inhibitors for the treatment of multiple inflammatory diseases. *European Journal of Medicinal Chemistry* **295** (2025).

19. Albanese, V. *et al.* Novel Aryl Sulfonamide Derivatives as NLRP3 Inflammasome Inhibitors for the Potential Treatment of Cancer. *J Med Chem* **66**, 5223-5241 (2023).

20. Li, J.M. *et al.* Scaffold Hybrid of the Natural Product Tanshinone I with Piperidine for the Discovery of a Potent NLRP3 Inflammasome Inhibitor. *Journal of Medicinal Chemistry* **66**, 2946-2963 (2023).

21. Xu, Y. *et al.* Development of sulfonamide-based NLRP3 inhibitors: Further modifications and optimization through structure-activity relationship studies. *Eur J Med Chem* **238**, 114468 (2022).

22. Sun, S. *et al.* Discovery of Novel 2,3-Dihydro-1H-indene-5-sulfonamide NLRP3 Inflammasome Inhibitors Targeting Colon as a Potential Therapy for Colitis. *J Med Chem* (2023).

23. Zhang, R. *et al.* New Highly Potent NLRP3 Inhibitors: Furanochalcone Velutone F Analogues. *ACS Med Chem Lett* **13**, 560-569 (2022).

24. Zhang, Z.W. *et al.* Design, Synthesis, and Bioevaluation of Novel NLRP3 Inhibitor with IBD Immunotherapy from the Virtual Screen. *Journal of Medicinal Chemistry* **67**, 16612-16634 (2024).

25. Zhao, M. *et al.* Novel Isoalantolactone-Based Derivatives as Potent NLRP3 Inflammasome Inhibitors: Design, Synthesis, and Biological Characterization. *J Med Chem* **67**, 7516-7538 (2024).

26. Li, N. *et al.* Discovery of Triazinone Derivatives as Novel, Specific, and Direct NLRP3 Inflammasome Inhibitors for the Treatment of DSS-Induced Ulcerative Colitis. *J Med Chem* **66**, 13428-13451 (2023).

27. Fulp, J. *et al.* Structural Insights of Benzenesulfonamide Analogues as NLRP3 Inflammasome Inhibitors: Design, Synthesis, and Biological Characterization. *J Med Chem* **61**, 5412-5423 (2018).

28. Yin, J. *et al.* NLRP3 Inflammasome Inhibitor Ameliorates Amyloid Pathology in a Mouse Model of Alzheimer's Disease. *Mol Neurobiol* **55**, 1977-1987 (2018).

29. Huang, C. *et al.* Discovery of novel biphenyl-sulfonamide analogues as NLRP3 inflammasome inhibitors. *Bioorg Chem* **146**, 107263 (2024).

30. Zhu, W.Q. *et al.* Peripheral Evolution of Tanshinone IIA and Cryptotanshinone for Discovery of a Potent and Specific NLRP3 Inflammasome Inhibitor. *Journal of Medicinal Chemistry* **68**, 3460-3479 (2025).

31. Sun, M. *et al.* Scaffold hopping-based structural modification of tranilast led to the identification of HNW005 as a promising NLRP3 inflammasome and URAT1 dual inhibitor for the treatment of gouty arthritis. *European Journal of Medicinal Chemistry* **292** (2025).

32. Zhang, X.N. *et al.* Design, synthesis and biological evaluation of phenyl vinyl sulfone based NLRP3 inflammasome inhibitors. *Bioorganic Chemistry* **128** (2022).

33. Xu, Y. *et al.* Design and Discovery of Novel NLRP3 Inhibitors and PET Imaging Radiotracers Based on a 1,2,3-Triazole-Bearing Scaffold. *J Med Chem* **67**, 555-571 (2024).

34. Yang, F. *et al.* Identification and Validation of PKR as a Direct Target for the Novel Sulfonamide-Substituted Tetrahydroquinoline Nonselective Inhibitor of the NLRP3 Inflammasome. *J Med Chem* **67**, 10168-10189 (2024).

35. Shao, J.J. *et al.* Britannin as a novel NLRP3 inhibitor, suppresses inflammasome activation in macrophages and alleviates NLRP3-related diseases in mice. *Acta Pharmacol Sin* **45**, 803-814 (2024).

36. Gastaldi, S. *et al.* Discovery of a novel 1,3,4-oxadiazol-2-one-based NLRP3 inhibitor as a pharmacological agent to mitigate cardiac and metabolic complications in an experimental model of diet-induced metaflammation. *Eur J Med Chem* **257**, 115542 (2023).

37. Lamkanfi, M. *et al.* Glyburide inhibits the Cryopyrin/Nalp3 inflammasome. *J Cell Biol* **187**, 61-70 (2009).

38. Hooftman, A. *et al.* The Immunomodulatory Metabolite Itaconate Modifies NLRP3 and Inhibits Inflammasome Activation. *Cell Metab* **32**, 468-478.e467 (2020).

39. Ambati, M. *et al.* Identification of fluoxetine as a direct NLRP3 inhibitor to treat atrophic macular degeneration. *P Natl Acad Sci USA* **118** (2021).

40. Liao, K.C. *et al.* Application of immobilized ATP to the study of NLRP inflammasomes. *Arch Biochem Biophys* **670**, 104-115 (2019).

41. Dai, Z. *et al.* Development of Novel Tetrahydroquinoline Inhibitors of NLRP3 Inflammasome for Potential Treatment of DSS-Induced Mouse Colitis. *J Med Chem* **64**, 871-889 (2021).

42. Lv, Q. *et al.* Dehydrocostus Lactone Effectively Alleviates Inflammatory Diseases by Covalently and Irreversibly Targeting NLRP3. *Medcomm* **6** (2025).

43. Sun, C. *et al.* Discovery, Total Synthesis, and Anti-Inflammatory Evaluation of Naturally Occurring Naphthopyrone-Macrolide Hybrids as Potent NLRP3 Inflammasome Inhibitors. *Angew Chem Int Ed Engl* **63**, e202405860 (2024).

44. Nan, J. *et al.* Discovery and structure-activity relationship studies of 3-pyridazinesulfonyl derivatives as a new class of inhibitors against NLRP3 inflammasome-dependent pyroptosis. *Eur J Med Chem* **296**, 117796 (2025).

45. Jiao, Y. *et al.* Discovery of a novel and potent inhibitor with differential species-specific effects against NLRP3 and AIM2 inflammasome-dependent pyroptosis. *European Journal of Medicinal Chemistry* **232** (2022).

46. Chen, Y. *et al.* Discovery and optimization of 4-oxo-2-thioxo-thiazolidinones as NOD-like receptor (NLR) family, pyrin domain-containing protein 3 (NLRP3) inhibitors. *Bioorg Med Chem Lett* **30**, 127021 (2020).

47. Jiang, H. *et al.* Identification of a selective and direct NLRP3 inhibitor to treat inflammatory disorders. *J Exp Med* **214**, 3219-3238 (2017).

48. Zhang, K. *et al.* Sorbremnoids A and B: NLRP3 Inflammasome Inhibitors Discovered from Spatially Restricted Crosstalk of Biosynthetic Pathways. *J Am Chem Soc* **146**, 18172-18183 (2024).

49. Marchetti, C. *et al.* OLT1177, a β-sulfonyl nitrile compound, safe in humans, inhibits the NLRP3 inflammasome and reverses the metabolic cost of inflammation. *Proc Natl Acad Sci U S A* **115**, E1530-e1539 (2018).

50. Cocco, M. *et al.* Electrophilic warhead-based design of compounds preventing NLRP3 inflammasome-dependent pyroptosis. *J Med Chem* **57**, 10366-10382 (2014).

51. Chen, L.Z. *et al.* Discovery of Novel Pterostilbene-Based Derivatives as Potent and Orally Active NLRP3 Inflammasome Inhibitors with Inflammatory Activity for Colitis. *J Med Chem* **64**, 13633-13657 (2021).

52. He, Y. *et al.* 3,4-methylenedioxy-β-nitrostyrene inhibits NLRP3 inflammasome activation by blocking assembly of the inflammasome. *J Biol Chem* **289**, 1142-1150 (2014).

53. Cocco, M. *et al.* Development of an Acrylate Derivative Targeting the NLRP3 Inflammasome for the Treatment of Inflammatory Bowel Disease. *J Med Chem* **60**, 3656-3671 (2017).

54. Gastaldi, S. *et al.* Chemical Modulation of the 1-(Piperidin-4-yl)-1,3-dihydro-2H-benzo[d]imidazole-2-one Scaffold as a Novel NLRP3 Inhibitor. *Molecules* **26** (2021).

55. Zhang, X., Hu, L., Xu, S., Ye, C. & Chen, A. Erianin: A Direct NLRP3 Inhibitor With Remarkable Anti-Inflammatory Activity. *Front Immunol* **12**, 739953 (2021).

56. Xu, H. *et al.* Costunolide covalently targets NACHT domain of NLRP3 to inhibit inflammasome activation and alleviate NLRP3-driven inflammatory diseases. *Acta Pharm Sin B* **13**, 678-693 (2023).

57. Huang, Y. *et al.* Tivantinib alleviates inflammatory diseases by directly targeting NLRP3. *iScience* **26**, 106062 (2023).

58. Zhou, Y. *et al.* Discovery of a selective NLRP3-targeting compound with therapeutic activity in MSU-induced peritonitis and DSS-induced acute intestinal inflammation. *Cell Mol Life Sci* **80**, 230 (2023).

59. He, H. *et al.* Oridonin is a covalent NLRP3 inhibitor with strong anti-inflammasome activity. *Nat Commun* **9**, 2550 (2018).

60. Pang, L., Liu, H., Quan, H., Sui, H. & Jia, Y. Development of novel oridonin analogs as specifically targeted NLRP3 inflammasome inhibitors for the treatment of dextran sulfate sodium-induced colitis. *Eur J Med Chem* **245**, 114919 (2023).

61. He, C. *et al.* Hit-to-Lead Optimization of the Natural Product Oridonin as Novel NLRP3 Inflammasome Inhibitors with Potent Anti-Inflammation Activity. *J Med Chem* **67**, 9406-9430 (2024).

62. Li, M. *et al.* Design, synthesis, and biological evaluation of oridonin derivatives as novel NLRP3 inflammasome inhibitors for the treatment of acute lung injury. *Eur J Med Chem* **277**, 116760 (2024).

63. Chen, Y. *et al.* RRx-001 ameliorates inflammatory diseases by acting as a potent covalent NLRP3 inhibitor. *Cell Mol Immunol* **18**, 1425-1436 (2021).

64. Li, W. *et al.* Discovery of alantolactone as a naturally occurring NLRP3 inhibitor to alleviate NLRP3-driven inflammatory diseases in mice. *Br J Pharmacol* **180**, 1634-1647 (2023).

65. Tang, P. *et al.* Chloranthalactone B covalently binds to the NACHT domain of NLRP3 to attenuate NLRP3-driven inflammation. *Biochem Pharmacol* **226**, 116360 (2024).

66. Zeng, Q. *et al.* Berberine Directly Targets the NEK7 Protein to Block the NEK7-NLRP3 Interaction and Exert Anti-inflammatory Activity. *J Med Chem* **64**, 768-781 (2021).

67. Li, Q. *et al.* Licochalcone B specifically inhibits the NLRP3 inflammasome by disrupting NEK7-NLRP3 interaction. *EMBO Rep* **23**, e53499 (2022).

68. Jin, X. *et al.* Entrectinib inhibits NLRP3 inflammasome and inflammatory diseases by directly targeting NEK7. *Cell Rep Med* **4**, 101310 (2023).

69. Haseeb, M. *et al.* Novel Small-Molecule Inhibitor of NLRP3 Inflammasome Reverses Cognitive Impairment in an Alzheimer's Disease Model. *ACS Chem Neurosci* **13**, 818-833 (2022).

70. Wang, L.B. *et al.* Discovery of novel biaryl urea derivatives against IL-1/3 release with low toxicity based on NEK7 inhibitor. *European Journal of Medicinal Chemistry* **283** (2025).

71. Jin, X.Y., Yang, Y.Q., Liu, D.D., Zhou, X.R. & Huang, Y. Identification of a covalent NEK7 inhibitor to alleviate NLRP3 inflammasome-driven metainflammation. *Cell Commun Signal* **22** (2024).

72. Sylvain, A. *et al.* A cereblon-based glue degrader of NEK7 regulates NLRP3 inflammasome in a context-dependent manner. *Cell Chem Biol* **32**, 955-968 (2025).

73. Li, B.Y. *et al.* Discovery and Development of NLRP3 Inhibitors Targeting the LRR Domain to Disrupt NLRP3-NEK7 Interaction for the Treatment of Rheumatoid Arthritis. *J Med Chem* **67**, 9869-9895 (2024).

74. Park, H. *et al.* Octyl gallate has potent anti-inflammasome activity by directly binding to NLRP3 LRR domain. *J Cell Physiol* **239**, e31196 (2024).

75. Kaneko, N. *et al.* KN3014, a piperidine-containing small compound, inhibits auto-secretion of IL-1β from PBMCs in a patient with Muckle-Wells syndrome. *Sci Rep* **10**, 13562 (2020).

76. Moasses Ghafary, S. *et al.* Identification of NLRP3(PYD) Homo-Oligomerization Inhibitors with Anti-Inflammatory Activity. *Int J Mol Sci* **23** (2022).

77. Shi, C. *et al.* Discovery of NLRP3 inhibitors using machine learning: Identification of a hit compound to treat NLRP3 activation-driven diseases. *Eur J Med Chem* **260**, 115784 (2023).

78. Shi, C. *et al.* Scaffold hopping-driven optimization for the identification of NLRP3 inhibitors as potential gout therapeutics. *European Journal of Medicinal Chemistry* **279** (2024).

79. Huang, Y. *et al.* Tranilast directly targets NLRP3 to treat inflammasome-driven diseases. *EMBO Mol Med* **10** (2018).

80. Zhang, X. *et al.* Discovery of novel 8-hydroxyquinoline derivatives as NLRP3 inflammasome inhibitors with therapeutic potential for inflammatory bowel disease. *Eur J Med Chem* **298**, 118023 (2025).

81. Shim, D.W. *et al.* BOT-4-one attenuates NLRP3 inflammasome activation: NLRP3 alkylation leading to the regulation of its ATPase activity and ubiquitination. *Sci Rep* **7**, 15020 (2017).

82. Juliana, C. *et al.* Anti-inflammatory compounds parthenolide and Bay 11-7082 are direct inhibitors of the inflammasome. *J Biol Chem* **285**, 9792-9802 (2010).

83. Yang, G. *et al.* Repurposing Auranofin, an Anti-Rheumatic Gold Compound, to Treat Acne Vulgaris by Targeting the NLRP3 Inflammasome. *Biomol Ther (Seoul)* **28**, 437-442 (2020).

84. Peng, L. *et al.* Scutellarin ameliorates pulmonary fibrosis through inhibiting NF-κB/NLRP3-mediated epithelial-mesenchymal transition and inflammation. *Cell Death Dis* **11**, 978 (2020).

85. Wu, X. *et al.* Discovery of a Novel Oral Proteasome Inhibitor to Block NLRP3 Inflammasome Activation with Anti-inflammation Activity. *J Med Chem* **65**, 11985-12001 (2022).

86. Chen, H. *et al.* Myricetin inhibits NLRP3 inflammasome activation via reduction of ROS-dependent ubiquitination of ASC and promotion of ROS-independent NLRP3 ubiquitination. *Toxicol Appl Pharmacol* **365**, 19-29 (2019).

87. Li, X. *et al.* MF-094, a potent and selective USP30 inhibitor, accelerates diabetic wound healing by inhibiting the NLRP3 inflammasome. *Exp Cell Res* **410**, 112967 (2022).

88. Ren, G.M. *et al.* Pharmacological targeting of NLRP3 deubiquitination for treatment of NLRP3-associated inflammatory diseases. *Sci Immunol* **6** (2021).

89. Yin, K. *et al.* Discovery of autophagy-tethering compounds as potent NLRP3 degraders for IBD Immunotherapy. *Eur J Med Chem* **275**, 116581 (2024).

90. Shi, F.L. *et al.* Dimethyl fumarate ameliorates autoimmune hepatitis in mice by blocking NLRP3 inflammasome activation. *Int Immunopharmacol* **108**, 108867 (2022).

91. Pan, H. *et al.* Wedelolactone facilitates Ser/Thr phosphorylation of NLRP3 dependent on PKA signalling to block inflammasome activation and pyroptosis. *Cell Prolif* **53**, e12868 (2020).

92. Ito, M. *et al.* Bruton's tyrosine kinase is essential for NLRP3 inflammasome activation and contributes to ischaemic brain injury. *Nat Commun* **6**, 7360 (2015).

93. Zhu, H. *et al.* Janus Kinase Inhibition Ameliorates Ischemic Stroke Injury and Neuroinflammation Through Reducing NLRP3 Inflammasome Activation via JAK2/STAT3 Pathway Inhibition. *Front Immunol* **12**, 714943 (2021).

94. Lin, W.Y. *et al.* Repositioning of the Angiotensin II Receptor Antagonist Candesartan as an Anti-Inflammatory Agent With NLRP3 Inflammasome Inhibitory Activity. *Front Immunol* **13**, 870627 (2022).

95. Liu, W. *et al.* A novel benzo[d]imidazole derivate prevents the development of dextran sulfate sodium-induced murine experimental colitis via inhibition of NLRP3 inflammasome. *Biochem Pharmacol* **85**, 1504-1512 (2013).

96. Sethi, G.S., Sharma, S. & Naura, A.S. PARP inhibition by olaparib alleviates chronic asthma-associated remodeling features via modulating inflammasome signaling in mice. *IUBMB Life* **71**, 1003-1013 (2019).

97. Cao, D.Y. *et al.* Discovery of a Covalent Inhibitor of Pro-Caspase-1 Zymogen Blocking NLRP3 Inflammasome Activation and Pyroptosis (vol 17, pg 15873, 2024). *Journal of Medicinal Chemistry* **67**, 20721-20721 (2024).

98. Youm, Y.H. *et al.* The ketone metabolite β-hydroxybutyrate blocks NLRP3 inflammasome-mediated inflammatory disease. *Nat Med* **21**, 263-269 (2015).

99. Liu, P. *et al.* Methyl Gallate Improves Hyperuricemia Nephropathy Mice Through Inhibiting NLRP3 Pathway. *Front Pharmacol* **12** (2021).

100. Hsieh, C.Y. *et al.* Synthetic 4-Hydroxy Auxarconjugatin B, a Novel Autophagy Inducer, Attenuates Gouty Inflammation by Inhibiting the NLRP3 Inflammasome. *Cells* **9** (2020).

101. Guo, W. *et al.* Small molecule-driven mitophagy-mediated NLRP3 inflammasome inhibition is responsible for the prevention of colitis-associated cancer. *Autophagy* **10**, 972-985 (2014).

102. Yue, K. *et al.* First-in-Class Hydrazide-Based HDAC6 Selective Inhibitor with Potent Oral Anti-Inflammatory Activity by Attenuating NLRP3 Inflammasome Activation. *J Med Chem* **65**, 12140-12162 (2022).

103. Yue, K.R. *et al.* Development of Hydrazide-Based HDAC6 Selective Inhibitors for Treating NLRP3 Inflammasome-Related Diseases. *Journal of Medicinal Chemistry* **68**, 9279-9302 (2025).

104. Li, C.G. *et al.* Baicalin Inhibits NOD-Like Receptor Family, Pyrin Containing Domain 3 Inflammasome Activation in Murine Macrophages by Augmenting Protein Kinase A Signaling. *Front Immunol* **8**, 1409 (2017).

105. Chen, S.Y. *et al.* Theaflavin mitigates acute gouty peritonitis and septic organ injury in mice by suppressing NLRP3 inflammasome assembly. *Acta Pharmacol Sin* **44**, 2019-2036 (2023).

106. Xu, J., Pickard, J.M. & Núñez, G. FDA-approved disulfiram inhibits the NLRP3 inflammasome by regulating NLRP3 palmitoylation. *Cell Rep* **43**, 114609 (2024).
